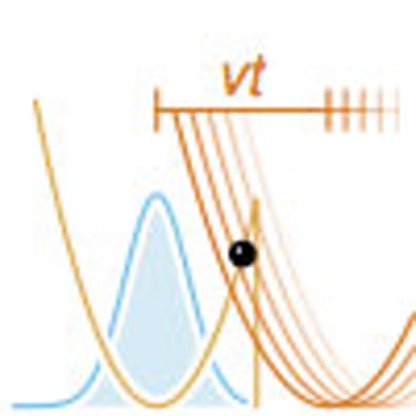# Theory of rapid force spectroscopy

**DOI:** 10.1038/ncomms5463

**Published:** 2014-07-31

**Authors:** Jakob T. Bullerjahn, Sebastian Sturm, Klaus Kroy

**Affiliations:** 1 Universität Leipzig, Institut für theoretische Physik, 04103 Leipzig, Germany

## Abstract

In dynamic force spectroscopy, single (bio-)molecular bonds are actively broken to assess their range and strength. At low loading rates, the experimentally measured statistical distributions of rupture forces can be analysed using Kramers’ theory of spontaneous unbinding. The essentially deterministic unbinding events induced by the extreme forces employed to speed up full-scale molecular simulations have been interpreted in mechanical terms, instead. Here we start from a rigorous probabilistic model of bond dynamics to develop a unified systematic theory that provides exact closed-form expressions for the rupture force distributions and mean unbinding forces, for slow and fast loading protocols. Comparing them with Brownian dynamics simulations, we find them to work well also at intermediate pulling forces. This renders them an ideal companion to Bayesian methods of data analysis, yielding an accurate tool for analysing and comparing force spectroscopy data from a wide range of experiments and simulations.

## Abstract (web summary)

Dynamic force spectroscopy is widely applied to probe molecular interactions by forcible bond breaking, but it currently lacks an analytical theory that spans the divide between experiment and simulation. Here, such a unified framework is developed and shown to be accurate for slow and fast loading.

## Graphical abstract

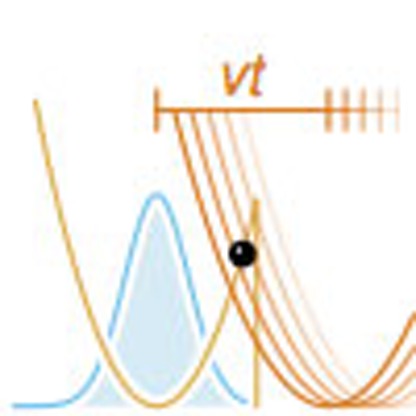

In the realm of soft matter, structural stability often hinges on delicate intermolecular bonds that are easily broken apart through external forces. The resulting malleability is characteristic of biological matter on practically all scales from single proteins^1^,^2^,^3^ over mesoscale cellular scaffold structures^4^ and individual cells^5^ to whole tissues^6^. Thanks to the development of a variety of nanomanipulation methods, intermolecular interactions can nowadays be investigated on a single-molecule level using a number of techniques commonly referred to as ‘dynamic force spectroscopy’^7^,^8^,^9^,^10^,^11^,^12^, allowing the experimentalist to isolate single binding sites and probe their strength by quickly and reliably inducing hundreds or thousands of unbinding events. The wealth of stochastic unbinding trajectories thus obtained is routinely analysed through a schematic but effective description of molecular bonds in terms of the attraction range *x*_b_ and the activation energy 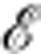 of the binding potential. To extract quantitatively useful predictions from this simple model, any theory of unbinding kinetics needs to provide a reasonable approximation to the underlying molecular dynamics. At the relatively low loading rates that were conventionally realized in experiments, any transient effects arising from the finite relaxation time of the bond itself can be neglected. This has allowed for the development of a range of analytical theories of forced bond breaking that greatly facilitate the analysis and interpretation of dynamic force spectroscopy data^13^,^14^,^15^,^16^,^17^,^18^. In contrast, detailed molecular dynamics simulations^2^ usually operate in the opposite limit of very high loading rates, where bond breaking becomes essentially deterministic^19^. Recently, also single-molecule assays are increasingly resolving the hitherto elusive rapid dynamics of bond breaking and accompanying macromolecular conformational changes, for example, in protein unfolding upon rapid loading^20^, or taut DNA recoil^21^ and supercoiling^22^. This provides a strong incentive to also push the existing theories to higher loading rates. Moreover, in spite of the experimental progress and improved computational abilities^23^, matching the loading rates used in experimental and simulation studies remains challenging^20^, making the development of a unified analytical theory of dynamic force spectroscopy, covering both fast and slow loading rates, all the more desirable.

To this end, we derive in the following a probabilistic theory of dynamic force spectroscopy in the spirit of Bell^24^, Evans & Ritchie^13^ and Dudko, Hummer & Szabo^14^ that becomes exact at high external forces (or, equivalently, high loading rates) and reduces to established results at low loading rates. We provide explicit, closed-form analytical results for the most common experimental loading protocols. They agree with exact numerical simulations of the microscopic bond model for all loading rates, save for a narrow region at the crossover from diffusion-dominated to deterministic dynamics. This makes them an ideal companion to Bayesian methods^25^ and a natural choice for the analysis of spectroscopy experiments and simulations alike. Moreover, we show that these results constitute the lowest-order approximation to a rigorous mathematical formulation of escape kinetics, which opens the way of their systematic extension to higher precision.

## Results

### Theory

Molecular binding and unbinding transitions lie at the heart of every chemical reaction and have thus been thoroughly investigated long before the advent of single-molecule manipulation techniques. In 1940, Kramers laid down a comprehensive, analytically tractable theory of chemical reaction rates^26^ that has since then become synonymous with reaction rate theory and can still be considered state of the art for most applications. The basic idea is that a molecular bond corresponds to a local free energy minimum and thus remains stable under weak perturbations. Its thermal fluctuations can be represented, within an effective picture, by those of a Brownian particle trapped in some one-dimensional binding potential *U*(*x*), with the ‘reaction coordinate’ *x* typically corresponding to the distance between two binding partners. The function *U*(*x*) should have a stable minimum at *x*=0, surrounded by a region of attraction extending to some finite coordinate *x*_b_>0 beyond which *U*(*x*) either vanishes or turns repulsive. As soon as the particle has left the basin of attraction, *x*(*t*)>*x*_b_, the bond can be considered broken. Excluding the possibility of quantum-mechanical tunnelling, which is negligible on a macromolecular scale, the dissociation process is a tug of war between thermal and potential forces. The thermal forces drive the bond coordinate *x* diffusively out of the bound state and the deterministic force *F*(*x*)=−*U*′(*x*) drags it back to the origin. On a probabilistic level, the dissociation rate of an ensemble of initially bound particles can then be deduced from an associated Fokker–Planck equation^27^, a partial differential equation for the time-dependent distribution of particles that is mathematically exact, but cannot be solved in closed form without the help of further simplifying assumptions. In most cases of practical interest, the activation energy 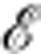 is large compared with the thermal energy scale, 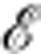
>>*k*_B_*T*. Dissociation then becomes a rare event, leaving sufficient time for any transients caused by the fast microscopic bond dynamics to die out. In the steady-state situation that ensues, an accurate analytical solution of the Fokker–Planck equation is possible^26^, yielding a time-independent unbinding rate *k* that scales exponentially in the activation energy, *k*∝exp(−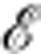/*k*_B_*T*).

This provides a natural starting point for the analysis of force-induced bond rupture. An external pulling force *F* acting along the reaction coordinate *x* ‘tilts’ the internal binding potential *U*, which lowers the effective free energy barrier and increases the unbinding rate. Within the Bell model^24^, the reaction distance *x*_b_ is assumed to remain fixed under the external force, such that the force-dependent unbinding rate immediately follows as *k*(*F*)=*k*(0)exp(*Fx*_b_/*k*_B_*T*). However, reaction rates are not measured directly in single-molecule experiments, where actual unbinding times may follow a broad statistical distribution. Also, external forces are usually not constant in time. In a typical experiment, molecular bonds are pulled apart using retracting actuators such as AFM cantilevers that exhibit Hookean stretching behaviour and therefore exert forces growing linearly with their retraction distance. Assuming an external force *F*(*t*) that steadily increases in time, Evans & Ritchie^13^ derived from the force-dependent unbinding rate *k*(*F*) a general expression for the experimentally measured distribution *p*(*F*) of rupture forces,


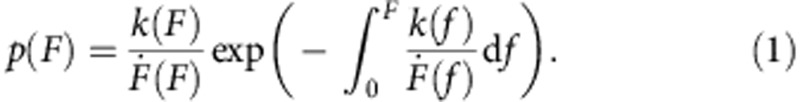


Here, the force dependence of the loading rate 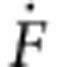 is optional, but can be used to take into account nonlinear loading protocols or nonlinearly elastic force transducers such as polymer linkers^28^,^29^. For a power-law binding potential and a cusp-shaped binding potential of finite range, Evans & Ritchie were furthermore able to improve upon the phenomenological Bell model by accounting for force-induced shifts in the reaction distance *x*_b_. Focusing on a linearly increasing external force, Dudko, Hummer & Szabo later derived analytic results for cusp-shaped^19^ and linear-cubic^30^ binding potentials. These could finally be condensed into a uniform expression for the rupture force distribution and its first two moments that contained a new fit parameter to smoothly interpolate between different potential shapes^14^,^15^. A recent crop of theories further fleshed out the free energy landscape by taking into account the force fluctuations pertaining to stiff actuators^16^,^17^,^18^ (that is, actuators with a spring constant 
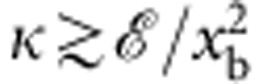
). These theories nowadays provide the standard tools used to convert rupture force histograms obtained from force spectroscopy experiments into estimates of the underlying binding potentials.

From a dynamical point of view, these approaches still rely on a strong separation between the short timescale of internal bond dynamics and the long timescale of bond dissociation. Although this approximation is well justified in the original Kramers theory^26^ of a force-free bond, it is bound to break down as soon as the external loading becomes rapid enough to effectively flatten out the energy barrier before rupture occurs. In that case, unbinding becomes essentially deterministic as the bond is swept away ballistically by the external force. As shown by Hummer & Szabo^19^ for a harmonic cusp potential, one can accurately predict the mean rupture force at high loading rates by neglecting stochastic fluctuations altogether and afterwards obtain a global approximation to the mean rupture force by manually interpolating between the high-force result and a conventional theory that accounts for the low-rate regime.

In the following, we derive a probabilistic theory of forced unbinding that includes fluctuations even at high pulling speeds and becomes exact both in the limits of high and low loading rates. Already the leading order results of this theory surpass previous work; moreover, it provides a systematic route for further improvement. We start with the same cusp-shaped binding potential as Hummer & Szabo,


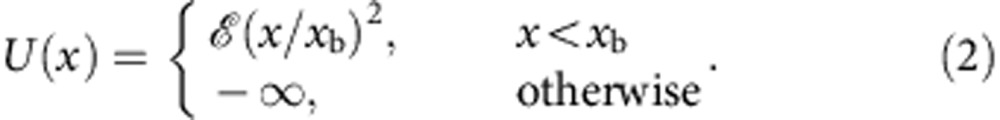


Formally, the potential becomes negatively infinite at *x*=*x*_b_, corresponding to an absorbing boundary condition (thus ignoring rebinding events, which quickly become negligible under external load^1^). Although it can be argued^14^ that a linear-cubic potential constitutes a more faithful approximation to many real binding potentials, we will show in the following that the practical difference between both models is often insignificant; what we lose in generality is more than made up for by what we gain in terms of analytical tractability.

To set the stage, we first summarize some basic definitions. The time-dependent probability *W*(*x*, *t*)d*x* to find any particle in the ensemble between *x* and *x*+d*x* follows from the Fokker–Planck equation^27^


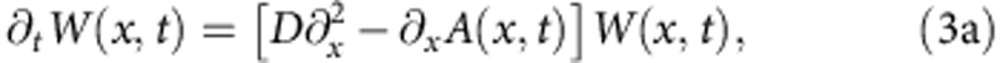



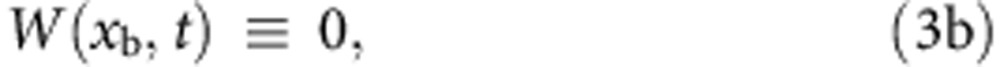



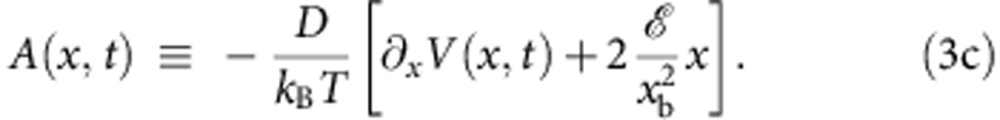


Here, *D* denotes the diffusion coefficient and *V*(*x*, *t*) an external pulling force potential that may either represent some prescribed force protocol *F*(*t*)≡ −∂_*x*_*V*(*x*, *t*) or a moving external spring, if *V*(*x*, *t*)≡*κ*[*x*−*y*(*t*)]^2^/2. The spatially integrated distribution


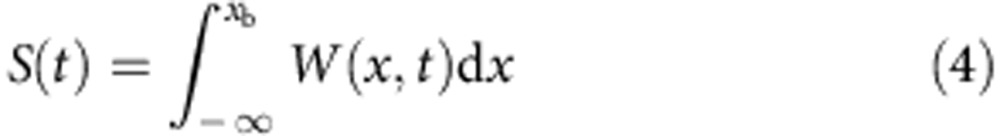


is the proportion of bound particles or ‘survival function’ at any given time *t*. A constant survival function *S*(*t*)=const corresponds to zero escape events and thus a vanishing unbinding rate *k*=0. Likewise, a positive escape rate *k*>0 amounts to an exponentially decaying survival function *S*(*t*)=*S*(0)exp(−*kt*). In the general case, *S*(*t*) may be neither constant nor exponential, but an arbitrarily complex function of time. However, it is always monotonously decaying and nonnegative and thus defines a (potentially time-dependent) generalized unbinding rate *k*(*t*),


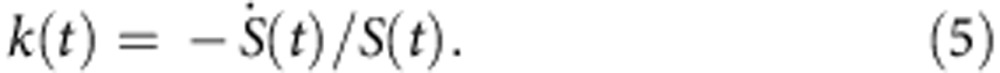


In principle, we could now solve equation (3a) (or an equivalent integral formulation^31^,^32^) numerically^33^ to obtain the time-dependent probability distribution *W*(*x*, *t*), calculate the survival probability *S*(*t*) via equation (4) and feed the result into equation (5) to precisely compute the unbinding rate *k*(*t*) for a known set of model parameters (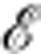, *x*_b_, *D*). This procedure is, however, too computationally intensive for the inverse problem of obtaining 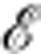, *x*_b_ and *D* from experimental data. To arrive at an analytical approximation to the unbinding rate that is quickly evaluated on a computer, we write down a first approximation to *W* by ignoring the absorbing boundary,


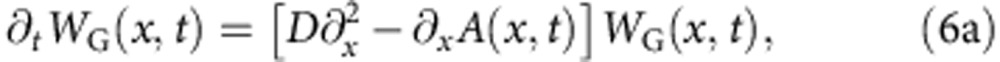



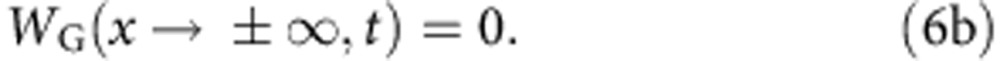


With the external driving potential *V*(*x*, *t*) in equation (3c), which is at most parabolic, *W*_G_ is strictly Gaussian for any localized initial distribution *W*_G_(*x*, *t*=0)=*δ*(*x*−*x*_0_). It is easily evaluated analytically,


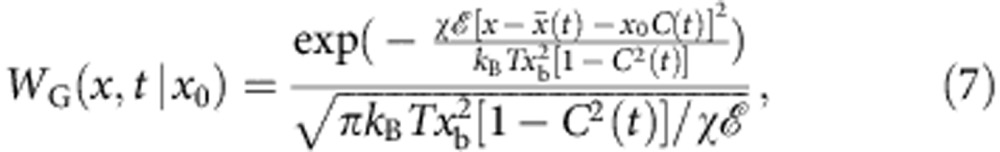


where 
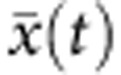
 follows from a purely deterministic equation of motion,


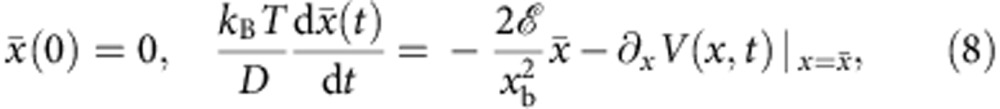


*C*(*t*) denotes the positional autocorrelation function,


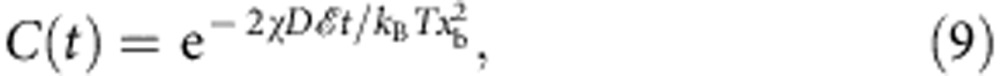


and χ the relative change in the system’s overall spring constant introduced by the external actuator; that is, *χ*=1 for external fields or soft springs 
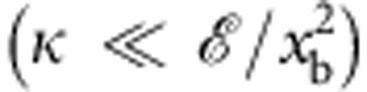
, 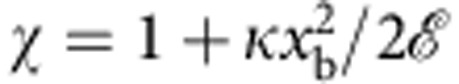 otherwise.

Going back to the time-dependent escape rate equation (5), we note that the computation of 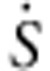
(*t*) via equation (4) would be needlessly complicated. Since escape events can only take place at the absorbing boundary *x*_b_, the outflow of bound particles into the unbound state is equivalent to the probability flux *j*(*x*_b_, *t*)= −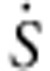
(*t*) across *x*_b_. It only depends on local properties of the probability distribution *W*, via the continuity equation


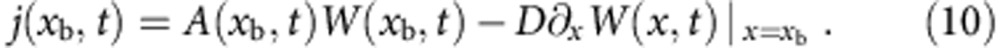


Replacing *W* by *W*_G_ in equation (10) defines the corresponding approximate flux *j*_G_, survival function 
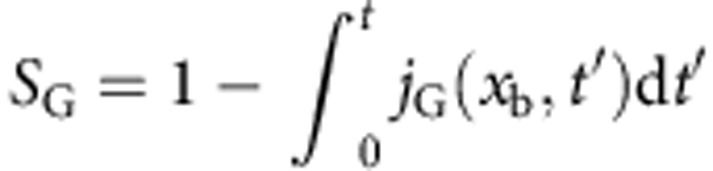
, and escape rate *k*_G_(*t*)=*j*_G_(*x*_b_, *t*)/*S*_G_(*t*).

Note that the Gaussian expressions become asymptotically exact for pulling forces greater than the critical force needed to flatten out the energy barrier, *F*>>*F*_c_=2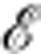/*x*_b_ (corresponding to high loading rates 
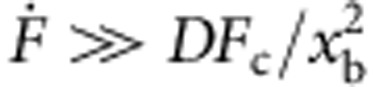
 in a constant-rate experiment), due to an essentially ballistic decay of the bound state. Intuitively, the probability density *W*_G_ does not have time to ‘sense’ (and react to) the presence of the absorbing boundary before it is pulled across *x*_b_ by the deterministic drift term *A*(*x*, *t*)*W*_G_(*x*, *t*). For the escape rate, we thus find the exact asymptotic limit


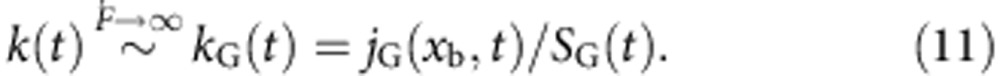


**Figure 1:**
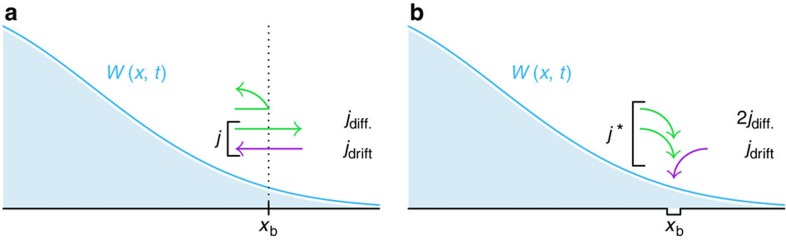
Flux into an absorber at *x*_b_. (**a**), Given some statistical distribution *W*(*x*, *t*) of particles, these particles can be driven across the position *x*_b_, either ballistically by external forces (*j*_drift_) or diffusively through random thermal noise (*j*_diff._). (**b**) Inserting an absorber at *x*_b_ effectively eliminates diffusive backscattering, thus doubling the diffusive contribution to the resultant probability flux j* into *x*_b_.

The situation is slightly more complicated for slow loading, where deterministic driving and stochastic dynamics interfere. Consider first the limiting case of a freely diffusing particle, that is, if the binding potential, the external forcing and the absorbing boundary condition are neglected. There is a purely diffusive net flux 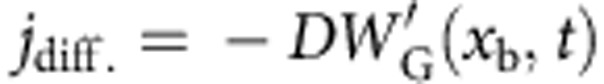 at the position *x*_b_, half of which is randomly backscattered (see Fig. 1). It is suggestive that inserting a perfect absorber at *x*=*x*_b_ would suppress the backscattering and therefore double the diffusive flux across *x*_b_. Adding the deterministic drift back in, we get


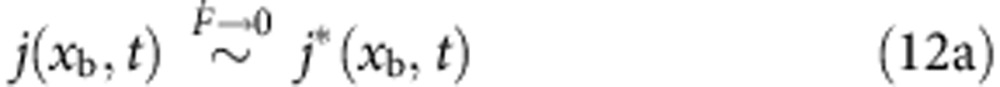



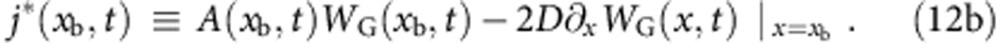


This expression for the flux can indeed be understood as the first-order approximation to an exact theory, as discussed in the Methods section. There we also show that j*(*x*_b_, *t*) provides an asymptotically exact expression for the escape rate at low loading rates,


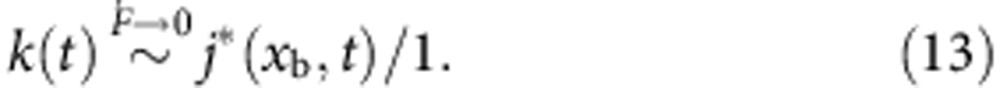


We now observe that the additional factor of two in front of the diffusive component in j* compared with *j*_G_ is irrelevant for the limit of large pulling rates, which is dominated by the deterministic drift term *A*(*x*_b_, *t*)*W*_G_(*x*_b_, *t*), suggesting that equation (11) still holds with *j*_G_ replaced by j*. Moreover, note that for high energy barriers 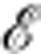
>>*k*_B_*T* and low pulling rates 
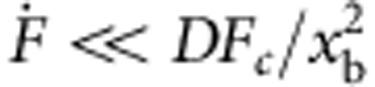
, the Gaussian survival probability *S*_G_(*t*) remains close to 1 since *W*_G_ extends only negligibly beyond *x*_b_ for all relevant times *t*, that is,


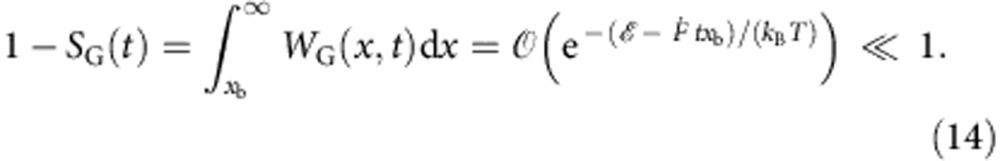


Our two asymptotic results can therefore be combined into the unified approximation


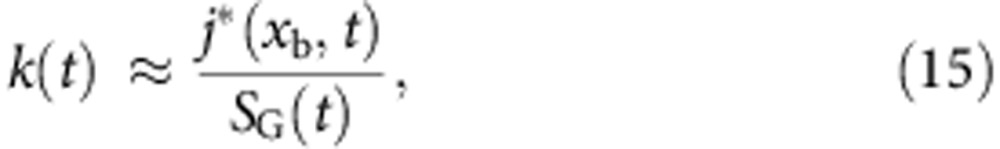


which is exact both in the limits of low and high loading rates. Together with equation (1), this yields the rupture force distribution


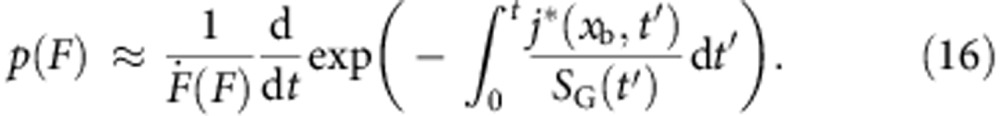


Evaluating *p*(*F*) in its general form equation (16) still requires some computational effort, since it depends in a potentially complicated way on experimental details such as the driving protocol, the initial distribution of particles within the bound state and the stiffness of the force actuator. However, for the common case of a linearly increasing pulling force *F*=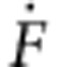
*t* (exerted by an external field or a soft spring with stiffness 
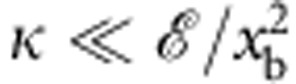
) we obtain the closed analytical result


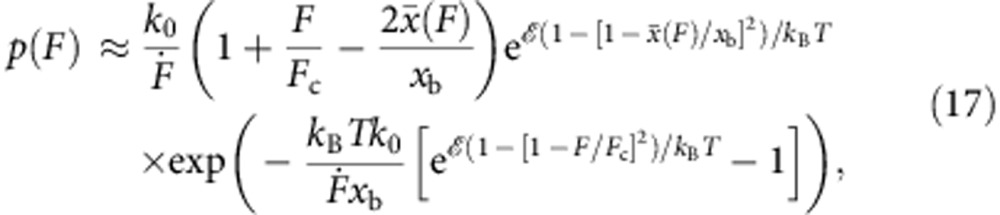


where *k*_0_ is the associated force-free Kramers rate for the cusp potential,


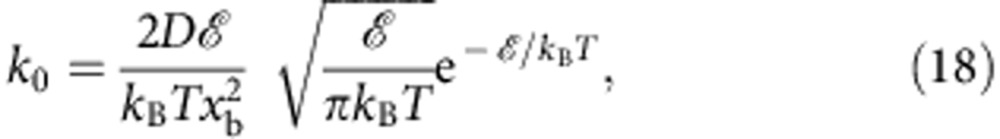


and 
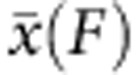
 is the force-dependent coordinate of the maximum of *W*_G_(*x*,*F*),


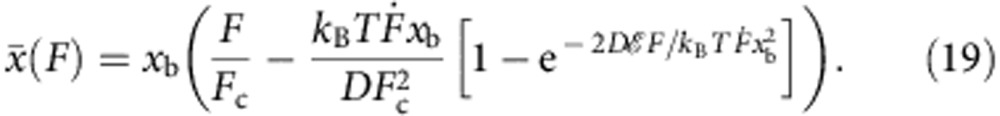


In the limit of small loading rates we obtain 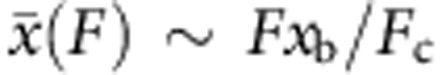 and equation (17) reduces to the expression derived by Dudko, Hummer & Szabo (DHS) in ref. 14 for *ν*=1/2. In the Methods section, we provide a compilation of analogous results for stiff force transducers, arbitrary driving protocols or arbitrary initial conditions *W*(*x*, *t*=0) (Table 1), as well as a closed expression for the mean rupture force ‹*F*›.

Some remarks about the comparison of data and theory may be useful, here. First, note that, in any case, a comparison of data and theory for the distribution *p*(*F*) is substantially more informative than merely fitting ‹*F*›. Yet, every analytical approximation to the true rupture force distribution depends on at least three different parameters (binding energy 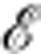, attraction range *x*_b_ and diffusivity *D*). Direct fits of experimentally obtained rupture force histograms are therefore prone to get trapped in some local optimum in parameter space, thus often missing the best possible solution. One way to obtain more reliable results is to first aggregate several data sets obtained under different loading rates and subsequently perform a ‘global’ fit using a single set of parameters only. Conventionally, this is done by either performing a least-squares fit of several histograms at once or by fitting the mean rupture force as a function of loading rate^13^,^14^,^15^,^19^. Both approaches can be improved upon systematically using a maximum-likelihood approach^25^ that, instead of optimizing for some arbitrary measure of fit quality (such as the squared residual), employs Bayesian analysis to select those model parameters most likely to underlie the given rupture force histograms. Compared with conventional fitting methods, the maximum-likelihood approach straightforwardly extends to heterogeneous data sets encompassing different linker stiffnesses or loading protocols. Moreover, it has proven significantly more robust with respect to small ensemble sizes^25^, a potentially crucial advantage in the analysis of real-world experiments. In general, the larger the range of applicability of a given theory of force spectroscopy, the better the results obtained using the maximum-likelihood approach. This fact should work to our advantage, as our model covers a much wider range of loading rates than the predominant Bell–Evans ‘standard model’^13^ and its recent extensions by DHS^14^ and others^16^,^17^,^18^. In the remainder and in the Supplementary Note 1, we illustrate the data fitting procedure with some examples and provide some practical tools and protocols for optimized data analysis.

### Application to simulation data

**Figure 2:**
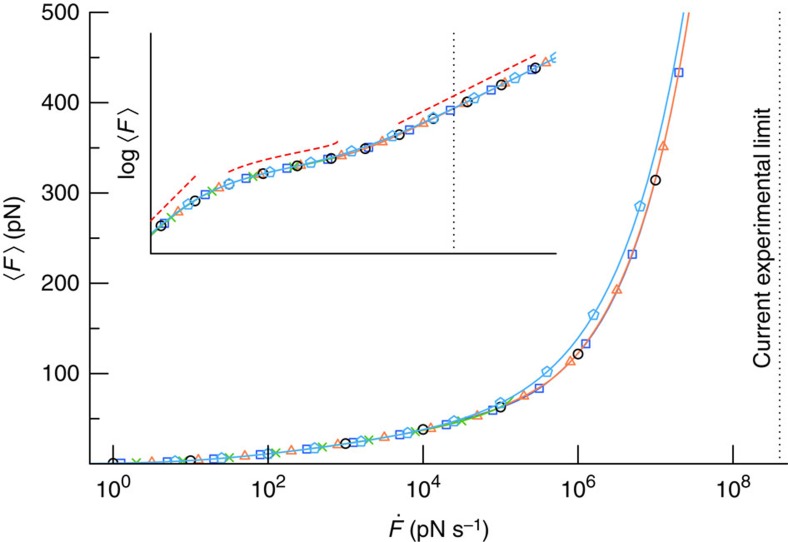
Mean rupture force ‹*F*› as a function of loading rate. Circles: ‹*F*› as determined from our numerical simulations (see Fig. 3). Crosses: best DHS^15^ fit (=9.49 × *k*_B_*T*, *x*_b_=1.01 nm, *D*=574 nm^2^ s^−1^). Triangles: best Hummer–Szabo^19^ fit (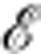
=10.2 × *k*_B_*T*, *x*_b_=1.0 nm, *D*=1015, nm^2^ s^−1^). Squares: ‹*F*› determined from *p*(*F*) (see Methods section), using the fit parameters obtained in Fig. 3. Pentagons: asymptotic analytical approximation equation (40) to ‹*F*›, using the fit parameters obtained in Fig. 3. Inset shows the same data in double-logarithmic coordinates. The mean rupture force converges onto the Hummer–Szabo^19^ asymptote 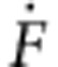
^1/2^ at large loading rates, 
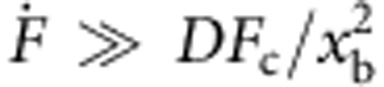
, and onto the logarithmic DHS asymptote ‹*F*›~[ln 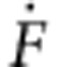]^1/2^ (ref. 14) at intermediate loading rates, 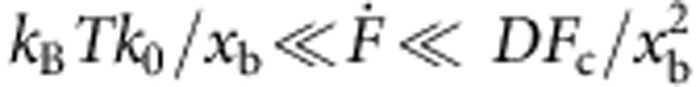 (dashed red lines, shifted upwards for better visibility). In the limit 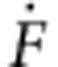
→0, external forces are too small to induce rupture, yielding as the measured rupture force the force at the time of spontaneous unbinding, ‹*F*›~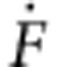/*k*_0_. For our choice of system parameters, the best currently available experimental setup^20^ would already be able to access the ballistic regime where ‹*F*› increases with 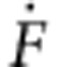
^1/2^.

**Figure 3:**
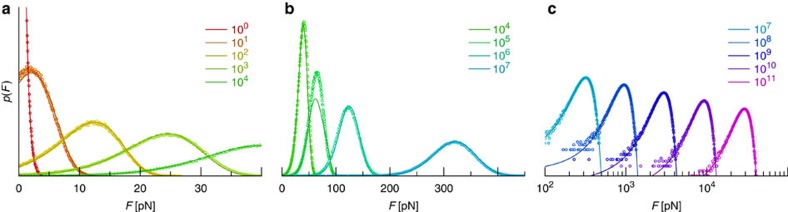
Analytical theory compared with simulations. Using a single set of model parameters, our theory (equation (17), solid lines) provides an accurate global approximation to both slow (**a**) and fast (**c**) external force protocols (as compared with the intramolecular relaxation timescale, 
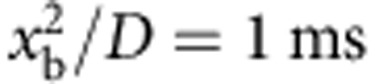
 in our case), apart from a narrow range (**b**) close to a critical loading rate (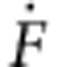
_c_≈10^5^ pN s^−1^ for our choice of parameters). The ‘experimental’ rupture force histograms have been generated by direct stochastic integration (see Methods section), using 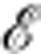
=10 × *k*_B_*T*, *T*=300 K, *x*_b_=1 nm, *D*=1,000 nm^2^ s^−1^ and 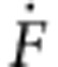
=1…10^11^ pN s^−1^. Our best-fit parameters obtained with equation (17) are =10.15 × *k*_B_*T*, *x*_b_=0.98 nm, *D*=976 nm^2^ s^−1^. Since the mean rupture force varies by orders of magnitude at large loading rates, we use double-logarithmic scaling for 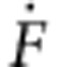
>10^7^ pN s^−1^ (**c**).

Although our theory becomes exact in the limits of high and low external loading rates, the quality of our approximation at finite loading rates is *a priori* unclear. To assess the practical applicability of our method, we have thus generated synthetic rupture force distributions using Brownian Dynamics simulations of the underlying microscopic bond model (see Methods). As Fig. 2 exemplifies, our theory is as good as the approach by Hummer & Szabo^19^ in capturing the mean rupture force ‹*F*› as a function of loading rate. Moreover, whereas the Hummer–Szabo model builds on an athermal treatment of the underlying microscopic equation of motion, we fully account for thermal fluctuations. These can be significant even within the ‘ballistic’ regime 
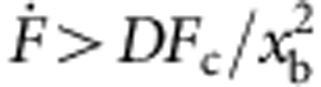
, as Fig. 3 and the experimental data by Rico *et al.*^20^ show. Hence, our approach allows us to perform a complete and systematic Bayesian analysis (see Methods and ref. 25) of rupture force histograms (as opposed to mean rupture forces), thus making full use of the available experimental data. As apparent from Fig. 3, our approximation still breaks down at intermediate loading rates, because, in our formalism, we do not actually absorb particles as they cross the absorbing boundary at *x*_b_ but merely count them. After leaving the potential well, they build up a ‘phantom population’ that may later produce an unphysical backflow into the bound state. In principle, this deficiency could be rectified, at least for the cusp potential, by extending the systematic integral equation approach outlined in the Methods to higher orders. Yet, the range of applicability of equation (16) already covers the vast majority of unbinding events. By simply truncating *p*(*F*) after its first zero crossing, we obtain an analytical expression for the rupture force distribution that works well at all loading rates, except for a narrow range close to a critical value 
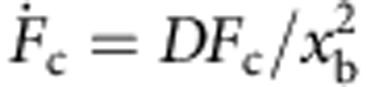
.

Although we find the derivation of an analytical, global approximation to the rupture force distribution *p*(*F*) satisfying in itself, its practical utility lies first and foremost in the accurate and efficient analysis of experimental unbinding data. To estimate how well our model stacks up against the prevailing methods of data analysis, we have thus analysed numerically obtained rupture force histograms, see Supplementary Fig. 1. We considered both the cusp-shaped potential and a linear-cubic binding potential (Supplementary Figs 2 and 3), using local and global (maximum-likelihood) fits both of equation (16) and of the rupture force distributions derived by DHS^14^ and Maitra & Arya^17^ (as well as a ‘cusp-optimized’ counterpart to their results, detailed in Supplementary Note 2) for soft and stiff linkers, respectively. Furthermore, we emulated conventional experimental procedures by fitting the mean rupture force *F*, using both the analytical expressions derived by Friddle^15^, associated to the DHS-model^14^, and Maitra & Arya^17^, as well as the numerical interpolation formula derived by Hummer & Szabo^19^. In the Supplementary Note 1, the interested reader will find a comprehensive discussion of all these approaches, the gist of which is as follows. At high loading rates, unsurprisingly, our theory proves superior to existing probabilistic theories^13^,^14^,^16^,^17^. More importantly, however, the combination of the maximum-likelihood analysis and our globally valid approximation is powerful enough to compete with the best conventional models even for a linear-cubic binding potential while requiring neither prior knowledge of the potential shape nor additional fit parameters. The only other global model to date, the numerical interpolation formula obtained by Hummer & Szabo, yields similarly good results as our own approach if supplied with sufficient data, although the increased computational cost of numerical integration leads to longer fitting times. Using a smaller range of loading rates (that is, 3–6 decades in 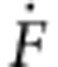 instead of 12), the higher information content of our full-histogram description can produce significantly better results. Moreover, as our approach inherits the greater generality and robustness of the Bayesian maximum-likelihood analysis, this gap is bound to widen with small ensemble sizes or heterogeneous data sets. Since the maximum-likelihood method is somewhat more difficult to implement in practice than traditional methods, we wrote (and provide for free use) a ready-made Mathematica notebook that covers some of the most common force spectroscopy setups, see Supplementary Data 1.

## Discussion

Our theory provides an analytically tractable generic model of dynamic force spectroscopy that reproduces previously known, accurate results^14^,^17^ at low loading rates and improves upon the results derived by Hummer & Szabo for high loading rates^19^ by including stochastic fluctuations. It provides excellent results also at intermediate loading rates, thus constituting an ideal companion to Bayesian methods of data analysis. Apart from serving as a systematic and convenient replacement for current theories of force spectroscopy, our approach may prove crucial to the analysis of future high-speed force spectroscopy setups and full-scale MD simulations. Finally, we note that at low enough loading rates, the effect of a polymeric force transducer onto the resultant rupture force distributions can be approximated by choosing *F*(*t*) and 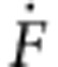
(*t*) accordingly^28^,^29^. At higher loading rates, the dynamics of force propagation within the polymer^21^,^34^,^35^ may become relevant, which could be an interesting subject for future theoretical developments based on our theory.

## Methods

### Alternative derivation of equation (12b)

The (non-Gaussian) probability distribution function *W*(*x*, *t*) is, in the presence of an absorbing boundary at *x*=*x*_b_, in general not analytically tractable. However, it is related to its Gaussian counterpart *W*_G_(*x*, *t*) in a simple manner^32^,^33^,


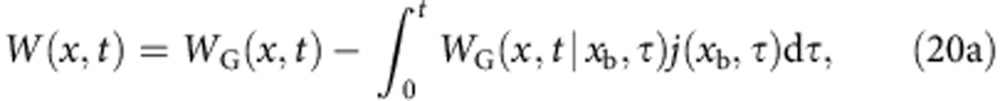



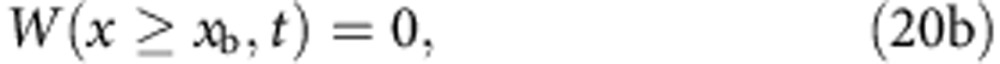


where *j*(*x*, *t*) is the associated probability flux, satisfying the continuity equation


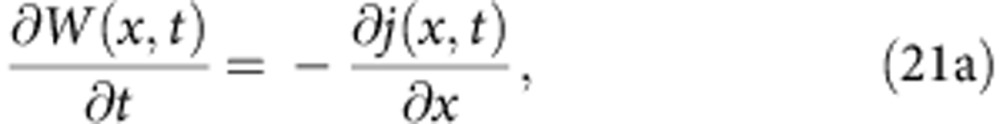


with the boundary conditions *W*(*x*, *t*)|_*x*→−∞_=*W*(*x*_b_, *t*)=0. The Gaussian distribution *W*_G_(*x*, *t*) and flux *j*_G_(*x*, *t*) also satisfy the continuity equation (21a) for *W*_G_(*x*, *t*)|_*x*→±∞_=0. Alternatively^36^, the absorbing boundary condition in equation (20b) can be interpreted as a sink term *σ*(*x*, *t*)<0 ∀*t*, resulting in the modified equation


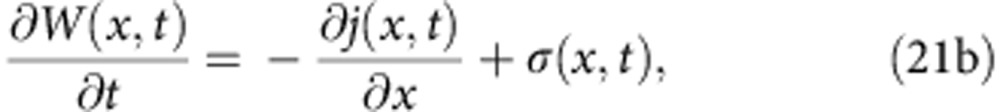


where *W*(*x*, *t*)|_*x*→±∞_=0. Using the fact that the flux vanishes at infinity, *j*(*x*→−∞, *t*)=0, we integrate equation (21a) to obtain


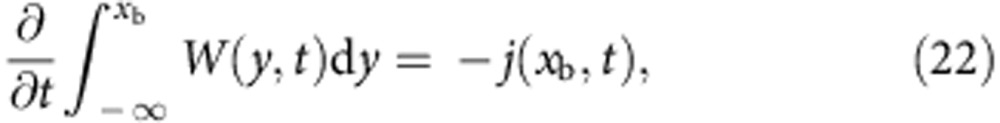


or equivalently, 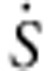
(*t*)=−*j*(*x*_b_, *t*).

Taking the time derivative of equation (20a) gives


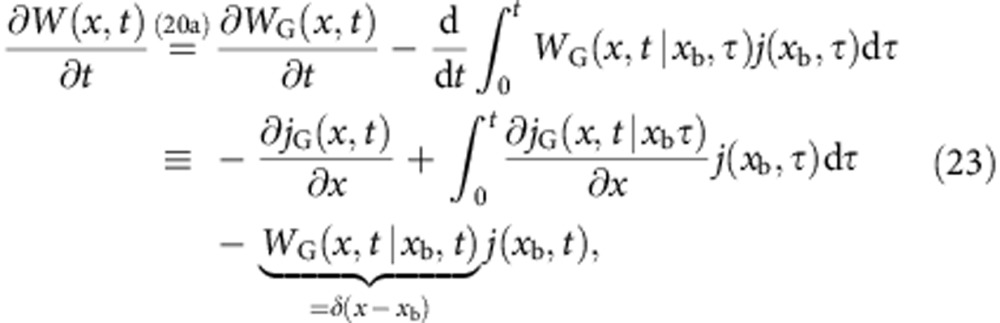


which can be compared with equation (21a), leading to


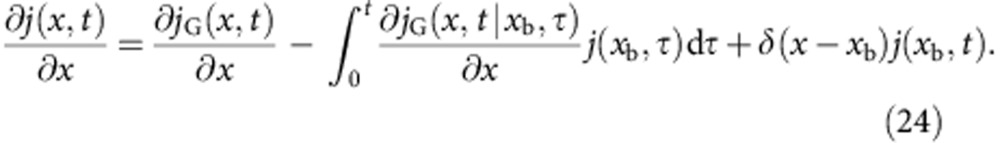


Inserting this expression into equation (21b) and then integrating over the interval (−∞, *x*_b_] results in


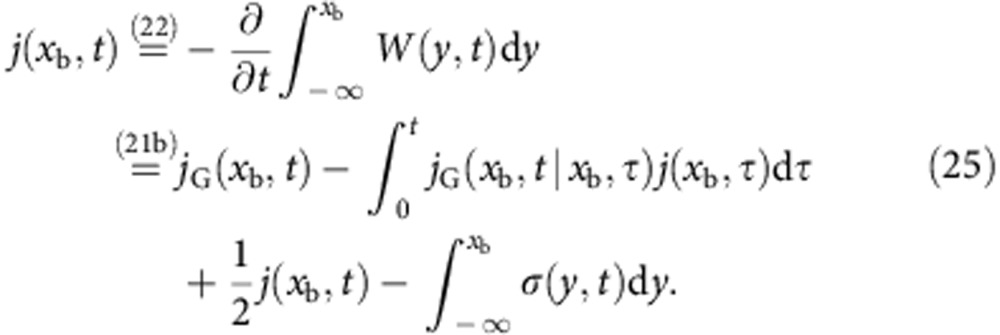


We now want to determine the sink term, *σ*(*x*,*t*). We shall therefore make the ansatz


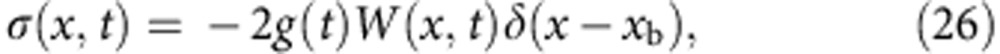


which, by introducing the effective flux *ψ*(*x*_b_, *t*)=*j*_G_(*x*_b_, *t*)+*g*(*t*)*W*_G_(*x*, *t*), reduces equation (25) to


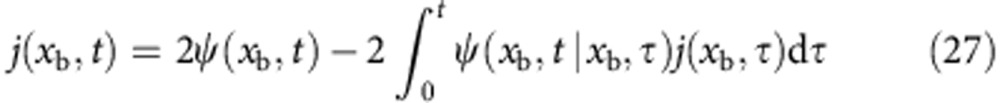


Equation (27) was originally derived by Buonocore *et al.* in ref. 37, where it was noted that the function *g*(*t*) can be uniquely determined using


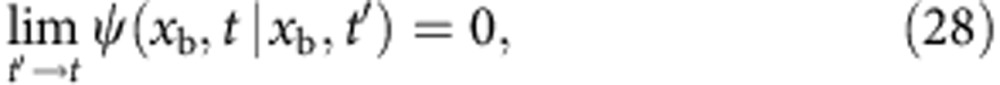


a criterion that follows from the fact that for all *t*,


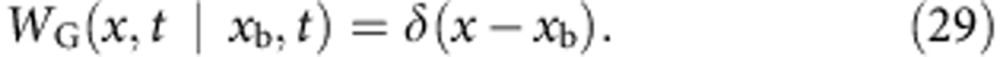


Solving equation (28), we obtain *g*(*t*)= −*A*(*x*_b_, *t*)/2 (ref. 38)^38^ and thus


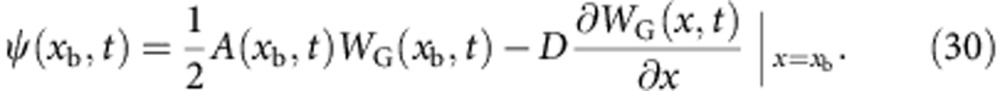


Formally, equation (27) can be solved iteratively,


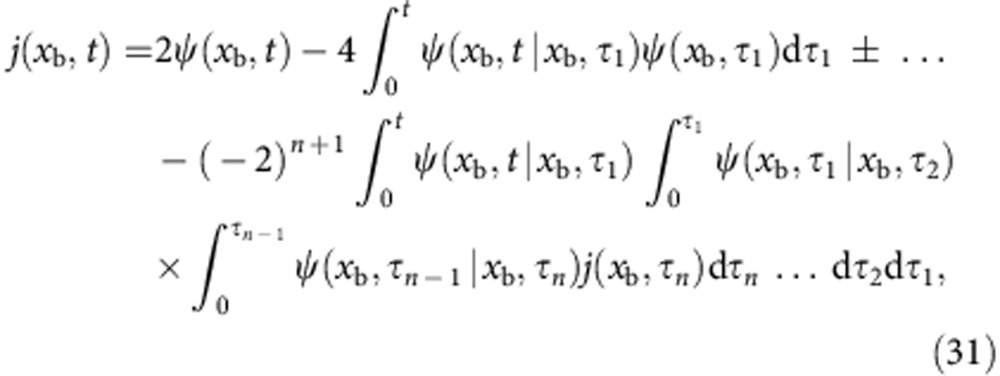


where 0<*τ*_*n*_<*τ*_*n*−1_<⋯<*τ*_1_<*t* and *j*(*x*_b_, *τ*_*n*_) vanishes as *n*→∞. Truncating the above result after the first term yields a first-order approximation *j*^0^(*x*_b_, *t*) to the flux that coincides with equation (12b),


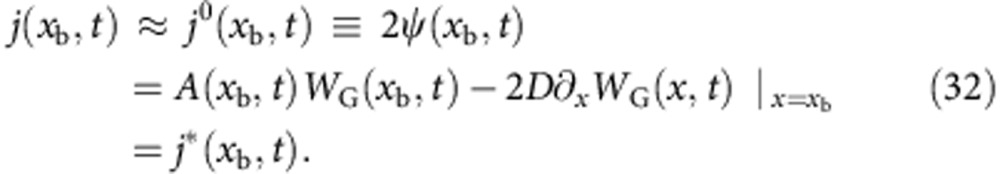


### Asymptotic exactness at low loading rates

Our escape rate *k*(*F*) and rupture force distribution *p*(*F*) both reduce to the results of DHS (equations 3 and 4 in ref. 14 for *ν*=1/2), in the limit 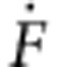
→0. The flux j*(*x*_b_, *F*) in equation (12b) is a sum of the Gaussian flux 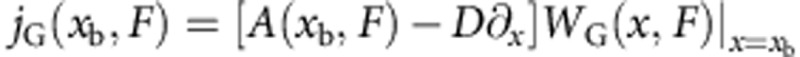 and the diffusive flux 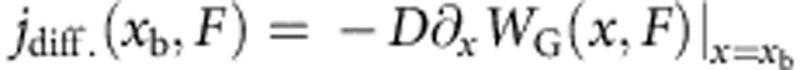. Due to their asymptotic behaviour,


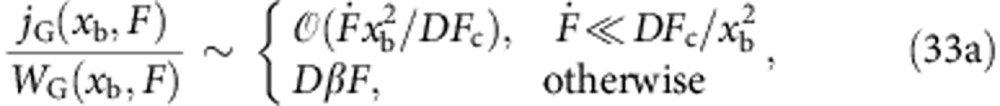



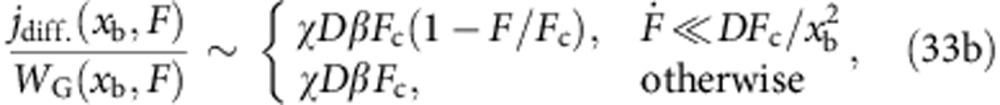


with *F*_c_=2/*x*_b_ and *F*(*t*) defined according to equation (53) for each limit, respectively, the diffusive flux dominates over the Gaussian flux at low loading rates. Using the expressions found in Table 1, we can determine the escape rate under the assumption that *S*(*F*)≈1 for 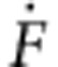
→0,


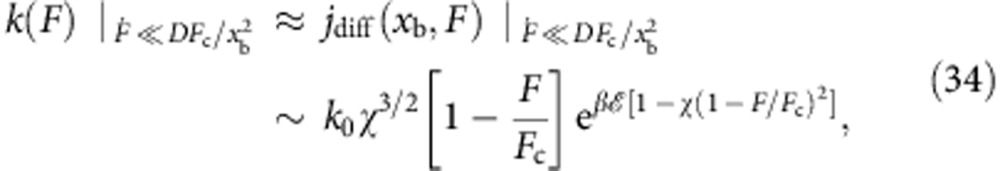


where *k*_0_ denotes the Kramers rate (equation 18). For *χ*=1 (that is, an external field or a soft spring, 
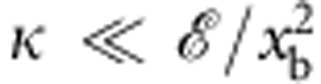
) the rate above coincides with equation 3 in ref. 14 for *ν*=1/2. For an external spring we have 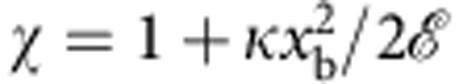 and obtain equation (3-S) of the Supplementary Note 2. Similarly, for 
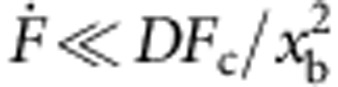
 it can be shown that *p*(*F*) reduces to equation 4 in ref. 14 for *ν*=1/2 in the case of an external field (*F*(*t*)=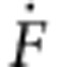
*t*) and to equation (4-S) for springs (*y*(*t*)=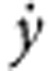
*t*), respectively.

### Mean rupture force

To compute the mean rupture force ‹*F*› shown in Fig. 2, we have eliminated the spurious zero crossings of *p*(*F*) at intermediate loading rates 
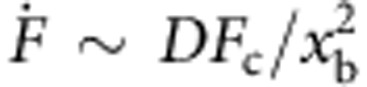
 by setting


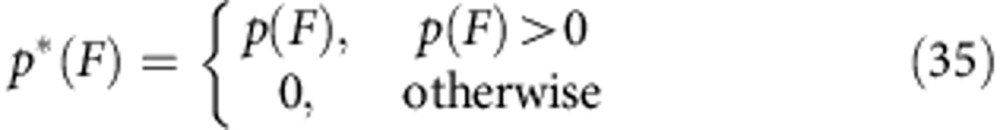


and computed from p*(*F*) the mean rupture force via


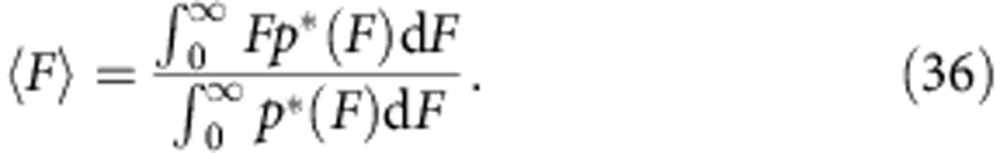


Alternatively, we can (for constant 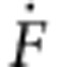 and *μ*=1) provide a rough analytical estimate that is exact in the limits of small and large loading rates, respectively,


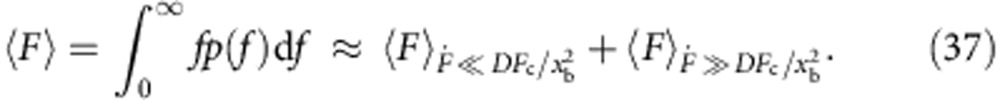


For large loading rates the mean rupture force evaluates to


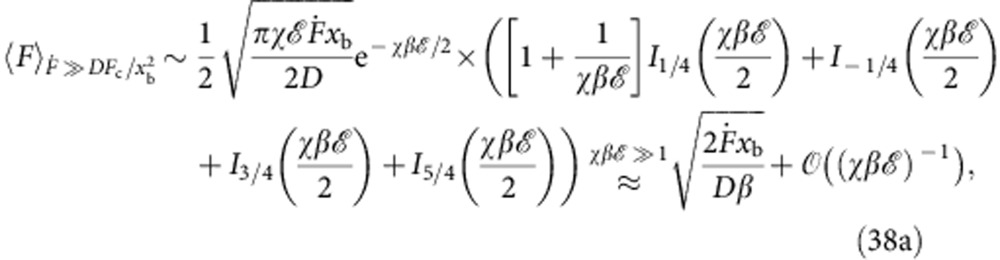


where *I*_*n*_(*z*) is the modified Bessel function of the first kind. This result coincides with the deterministic mean rupture force originally derived in ref. 19.

For small loading rates *F*≪*F*_c_, we arrive at a result previously obtained from the Bell–Evans model by Gergely *et al.*^39^,


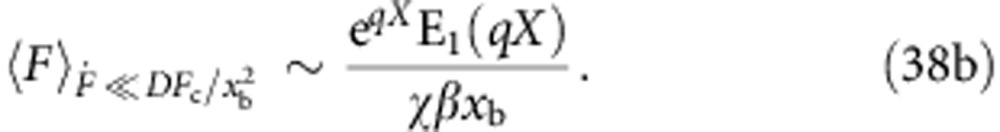


Here, *q*=exp(*β*
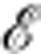
[1−*χ*]), *X*=*χ*^1/2^*k*_0_/*β*
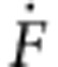
*x*_b_ and *E*_1_(*z*) denotes the exponential integral,


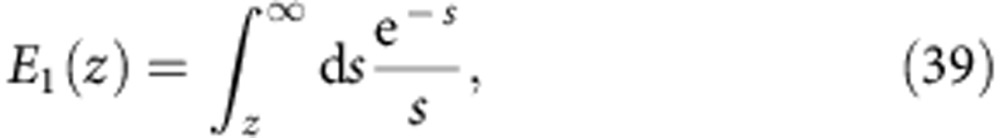


Inserting equation (38a) and (38b) into equation (37), we thus end up with


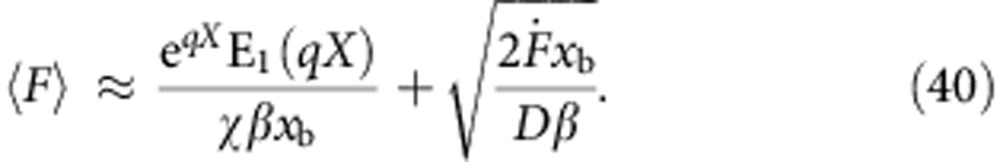


It should be noted that we provide ‹*F*› only for the sake of completeness. In fact, it is wasteful to discard experimentally measured force fluctuations instead of directly fitting the full histograms *p*(*F*) and we thus advise against the use of equation (40) (as much as any other available predictions for ‹*F*›) for analysing the experimental data.

### Simulations

To generate rupture force histograms across a large range of external loading rates, we have used a stochastic Euler scheme to directly integrate the equivalent Langevin equation for the Fokker–Planck equation (3a),


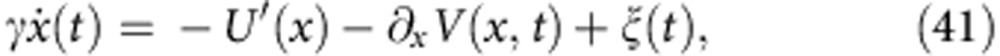


where *ξ*(*t*) denotes Gaussian, white noise,


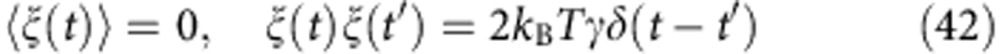


and *γ* the bond friction coefficient, *γ*=*k*_B_*T*/*D*. External force is applied either through an external field *V*(*x*,*t*)= −*x*
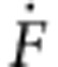
*t* (corresponding to a deterministic force) or through a moving external spring centred at *y*(*t*)=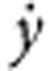
*t*. Initial particle positions are drawn from a Boltzmann distribution; once the particle position *x*(*t*) crosses *x*_b_, we consider the bond broken and record the corresponding rupture force. Although one might argue that prior knowledge of the barrier position *x*_b_ allowed us an unrealistic advantage over actual experiments, we note that even beyond the critical pulling force, strong variations in the intramolecular binding potential *U* around *x*_b_ manifest themselves in experimentally detectable force signatures, see Supplementary Note 3 and the accompanying Supplementary Fig. 4.

### Maximum-likelihood method

Following ref. 25, the likelihood *P* of a given set of parameters 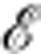, *x*_b_, *D* is computed as follows,


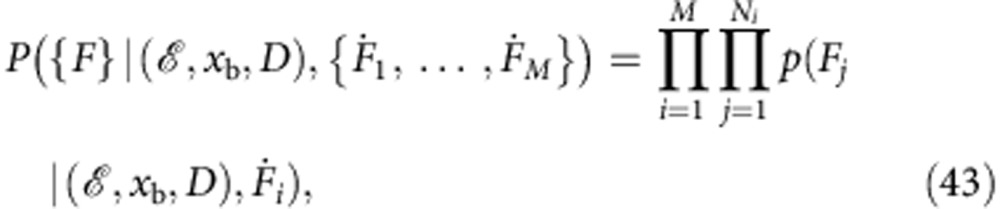


where {*F*} denotes the totality of rupture forces taken into consideration and


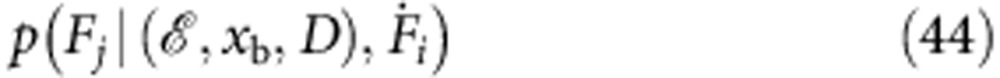


the rupture force distribution *p* given by equation (16), evaluated at the measured rupture forces *F*=*F*_*j*_, with model parameters (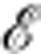, *x*_b_, *D*) and 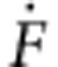
=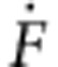
_*i*_. The logarithm of the right-hand side is then maximized using the ‘NMaximize’ method provided by Wolfram Mathematica. Since the maximum-likelihood method is somewhat more involved than conventional fitting procedures, we provide a ready-made Mathematica notebook that allows the user to analyse her data through a simple graphical interface (Supplementary Data 1).

### Nonconstant loading rates and arbitrary initial conditions

In deriving equation (17), we have started from a localized initial condition *W*(*x*,*t*=0)=*δ*(*x*−*x*_0_), calculated the corresponding distribution of first passage times and in the end averaged over all possible starting points *x*_0_<*x*_b_ using an equilibrium Boltzmann distribution. This choice of initial condition should be appropriate to most real-world scenarios, but since our theory readily extends to nonequilibrium initial conditions (corresponding, for example, to a particle held fixed by an auxiliary trapping potential that is turned off at *t*=0), Table 1 provides a generic rupture force distribution for arbitrary choices of *W*(*x*, *t*=0)≡*W*_0_(*x*). Importantly, for a thermally stable, smoothly varying binding potential *U* the initial distribution is generally Gaussian around the energy minimum at *x*=0,


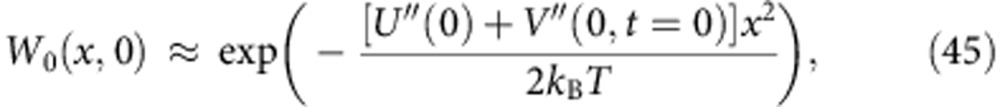


but its width may deviate from the value obtained from our cusp-shaped model potential. To better account for other types of binding potentials (such as the often used linear-cubic potential), we can thus treat


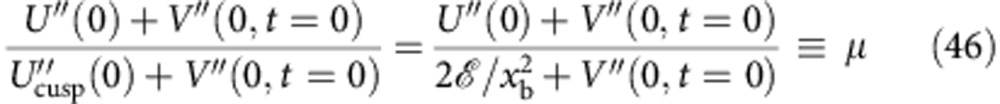


as an additional fit parameter, in analogy to the parameter *ν* used by Dudko, Hummer & Szabo^14^ (although in contrast to *ν*, our fit parameter *μ* is relevant only at high loading rates 
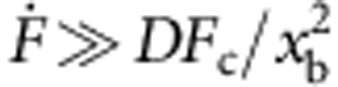
 since at low loading rates the memory of the initial condition fades away long before rupture occurs).

In Table 1, we also provide a generic expression for arbitrary force protocols *F*(*t*) that do not necessarily scale linearly in time.

### Stiff force transducers

For stiff transducers, that is, external spring constants 
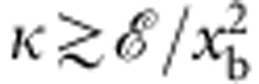
, the relation between rupture time and rupture force depends to a certain degree on the experimental time resolution. Instead of a deterministic time-dependent pulling force *F*(*t*), one measures then a force within the transducer that fluctuates with the bond coordinate *x*(*t*). These fast fluctuations are typically smoothed out using a time-moving average, which in a conventional low-speed experiment corresponds to an equilibrium average, with respect to the time-dependent combined potential *U*(*x*)+*V*(*x*, *t*) (molecular bond+transducer), yielding thus as the measured rupture force


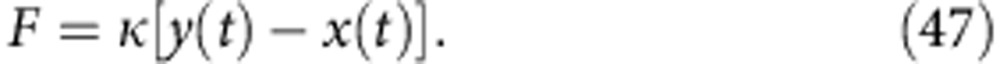


**Figure 4:**
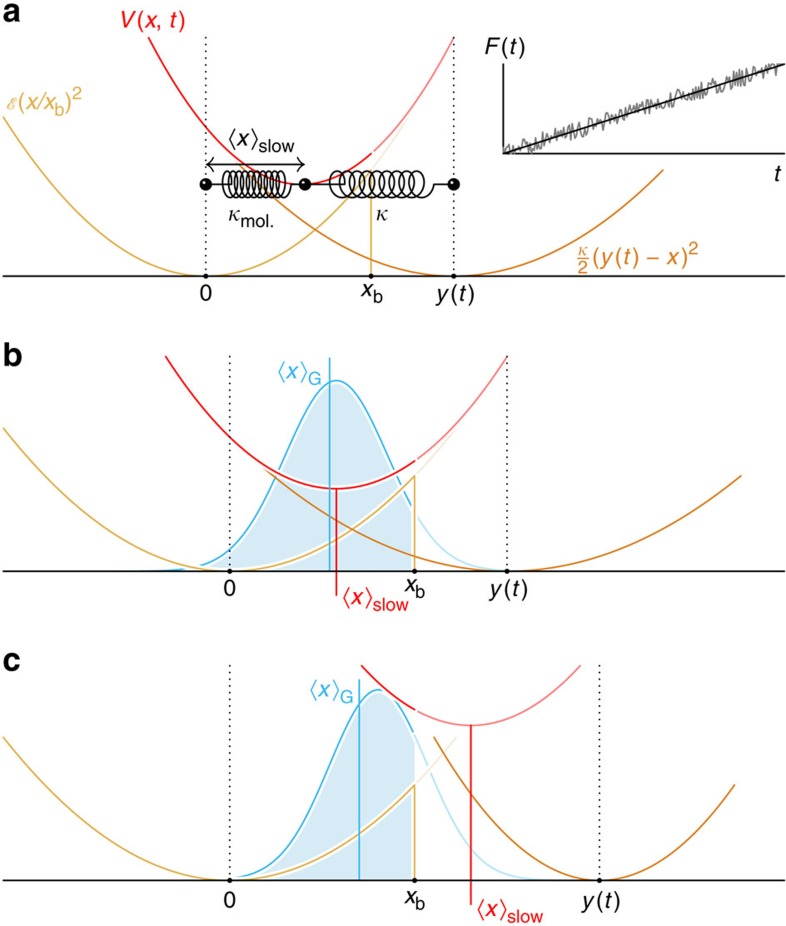
Measuring rupture forces with a stiff transducer. (**a**) As long as the bond remains intact, the combined bond-transducer system can be seen as two harmonic springs connected in series. At low pulling speeds, positional fluctuations within the bound state translate into force fluctuations that can be smoothed out via a time-moving average (see inset). (**b**) As long as the effective free energy barrier is still large compared with *k*_B_*T*, the probability distribution *W*(*x*, *t*) closely approximates a Gaussian centred within the bound state and ‹*x*›_slow_(*t*) virtually coincides with ‹*x*›_G_(*t*). (**c**) At high pulling forces, the static force-balance argument **a** fails, as it yields an equilibrium position ‹*x*›_slow_(*t*) beyond *x*_b_. The improved approximation ‹*x*›_G_(*t*) instead is always bounded above by *x*_b_.

As long as the effective free energy barrier is still large compared with *k*_B_*T* (which can safely be assumed in the limit of low loading rates 
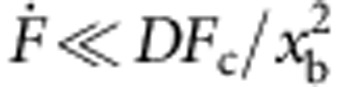
), the actual distribution of bound particles is essentially a Gaussian centred within the bound state. Hence, the anharmonicity of the molecular binding potential beyond *x*_b_ can be neglected and the equilibrium position ‹*x*› of bound particles follows from a simple force-balance argument^17^ (see Fig. 4a),


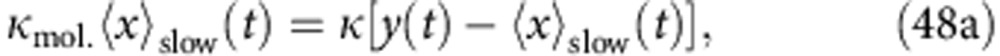



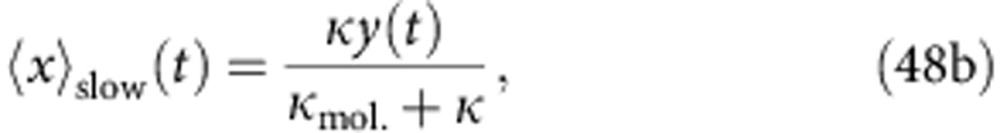


where 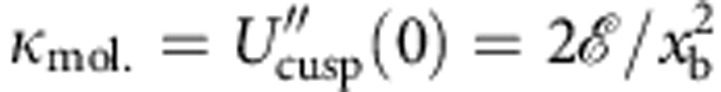 for the cusp potential. Setting 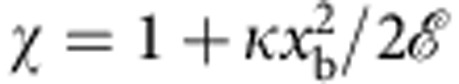, this simplifies to


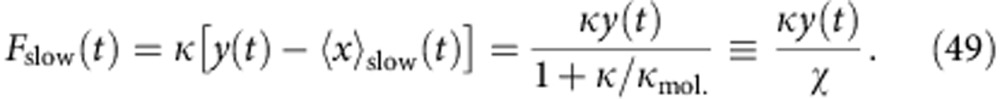


Large external forces, on the other hand, may shift ‹*x*›_slow_(*t*) beyond *x*_b_, rendering the static force-balance argument obviously inadequate. We obtain a reasonable generalization of equation (48b) by instead truncating the time-dependent approximate probability distribution *W*_G_(*x*, *t*|*x*_0_) at *x*=*x*_b_ and using that to compute ‹*x*›(*t*),


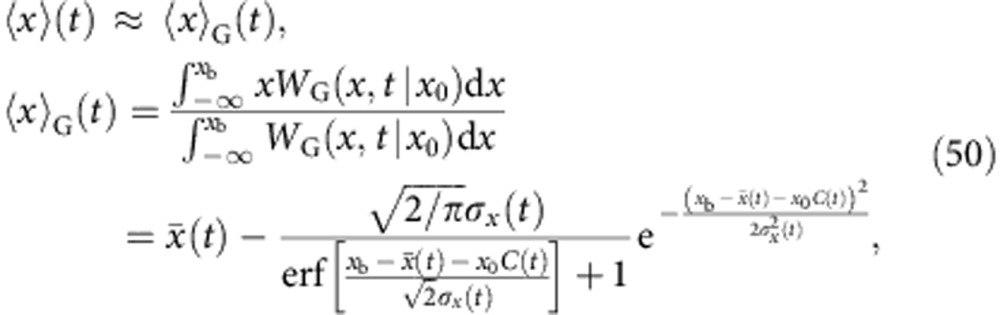


with mean 
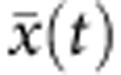
, variance 
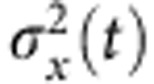
 and the autocorrelation function *C*(*t*) defined as usual (note that the particle position *x*_0_ at *t*=0 is irrelevant in the low-force limit and is eliminated in the high-force limit by averaging over the initial distribution of particle positions *W*(*x*_0_, *t*=0)),


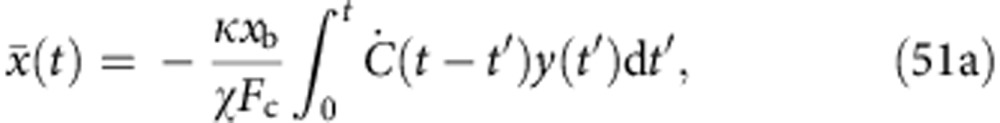



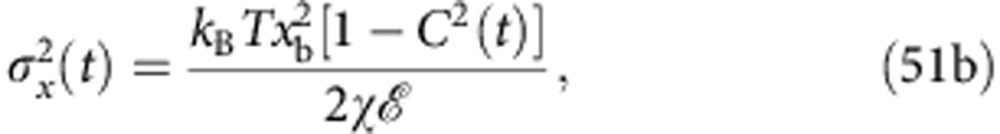



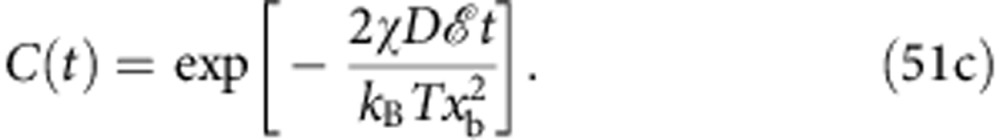


In the limit of low pulling forces, equation (50) reduces to the established result equation (48b), 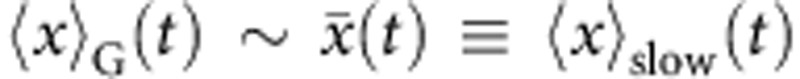. In the high-force limit, however, an equilibrium average is no longer warranted as a force spectroscopy setup with sufficient time resolution should instead measure the transducer force at the very moment of rupture,


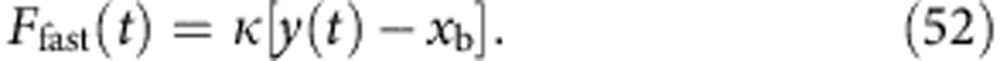


This equation coincides with the operational definition of ‘rupture force’ used by Hummer & Szabo^19^. Since in the limit of high loading rates, *x*_b_ is generally negligible compared with the transducer position *y*(*t*) at the moment of rupture and since ‹*x*›_G_(*t*) is bound above by *x*_b_, we can use *κ*[*y*(*t*)−‹*x*›_G_(*t*)] as a global approximation to the rupture force; the disadvantage of this approach is that *F*(*t*) becomes difficult to solve for *t*. At intermediate pulling speeds, it may be reasonable to perform the functional inversion numerically; in practice, however, we propose to simply replace *F*(*t*) by its respective asymptotic limits,


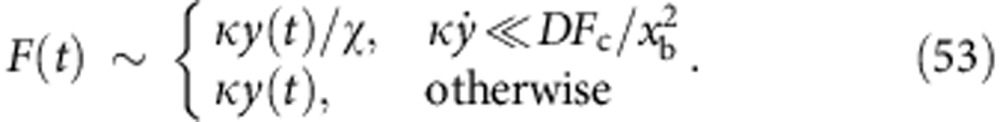


## Author contributions

J.T.B. and S.S. developed the theory and analysed the simulation data. S.S. and K.K. wrote the paper. J.T.B. and S.S. contributed equally to the study. All authors discussed the results and commented on the manuscript.

## Additional information

**How to cite this article:** Bullerjahn, J. T. *et al.* Theory of rapid force spectroscopy. *Nat. Commun.* 5:4463 doi: 10.1038/ncomms5463 (2014).

## Supplementary Material

Supplementary Figures and Notes Supplementary Figures 1-4 and Supplementary Notes 1-3

Supplementary Data 1 Mathematica Notebook for data analysis and User's Guide accompanying Mathematica Notebook

## Figures and Tables

**Table 1:** Analytical approximations to the rupture force distribution *p*(*F*).

**Figure:**
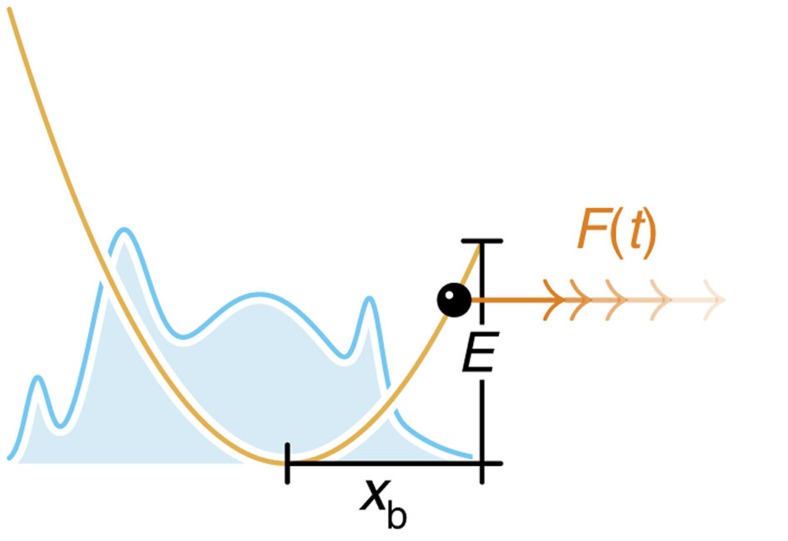


**Figure:**
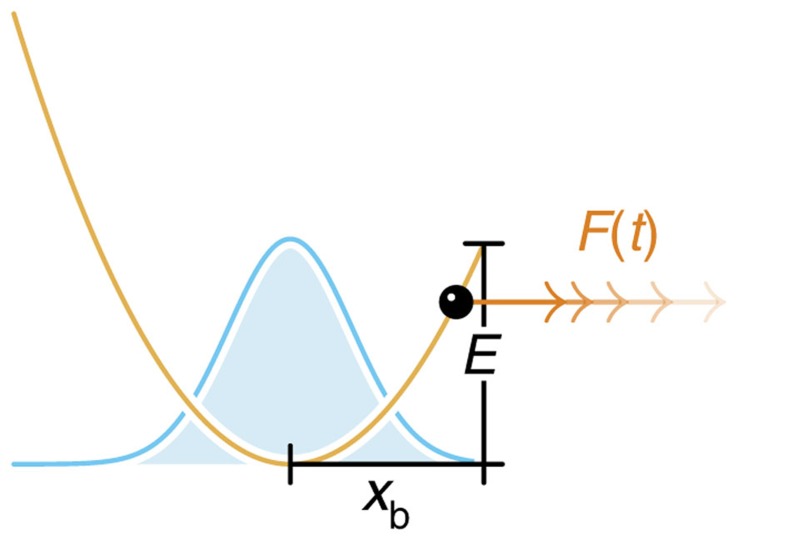


**Figure:**
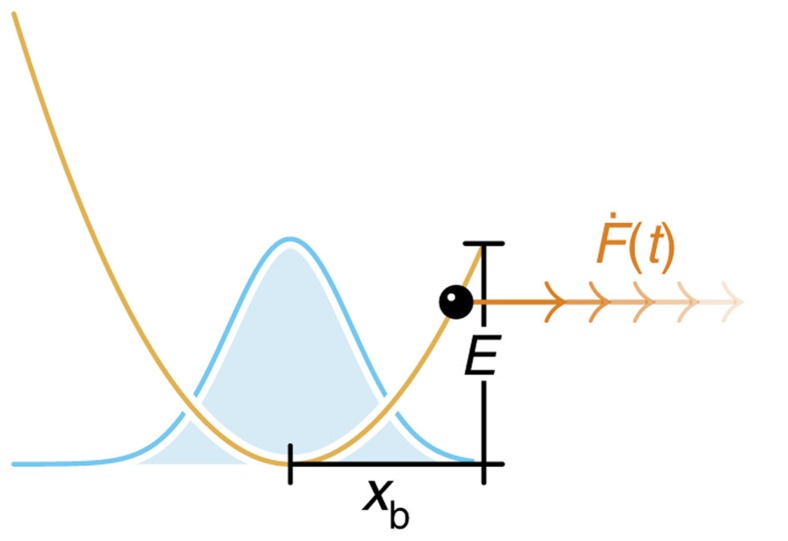


**Figure:**
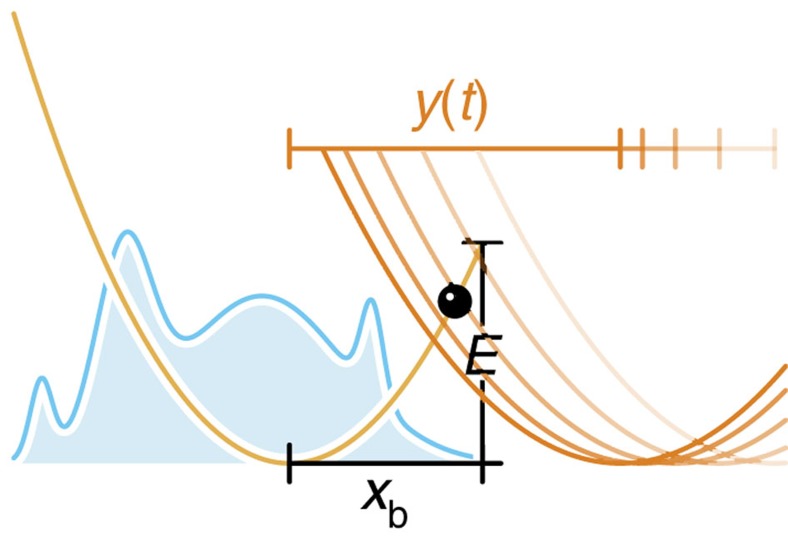


**Figure:**
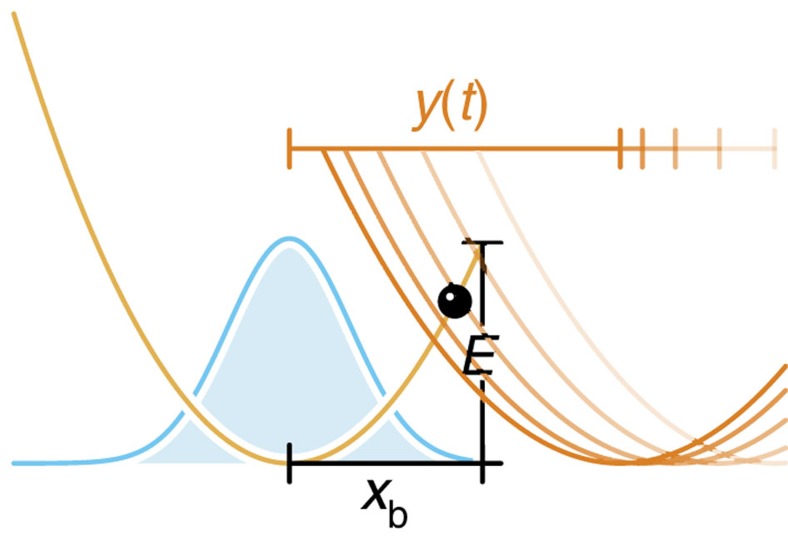


**Figure:**
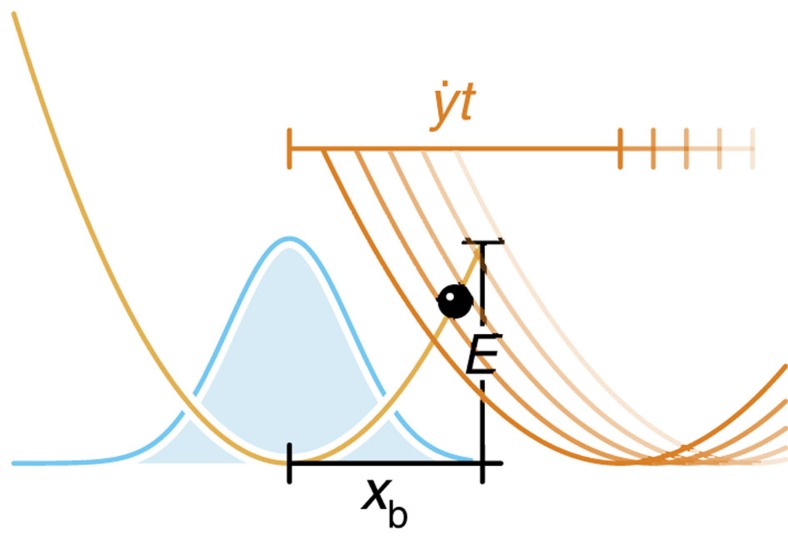


**Figure:**