# Supplementary material for: Theory of rapid force spectroscopy
Source: Nat Commun. 2014 Jul 31;5:4463. doi: 10.1038/ncomms5463 (PMC4124868; doi:10.1038/ncomms5463)
Supplement: Supplementary Figures and Notes — Supplementary Figures 1-4 and Supplementary Notes 1-3 [file ncomms5463-s1.pdf]

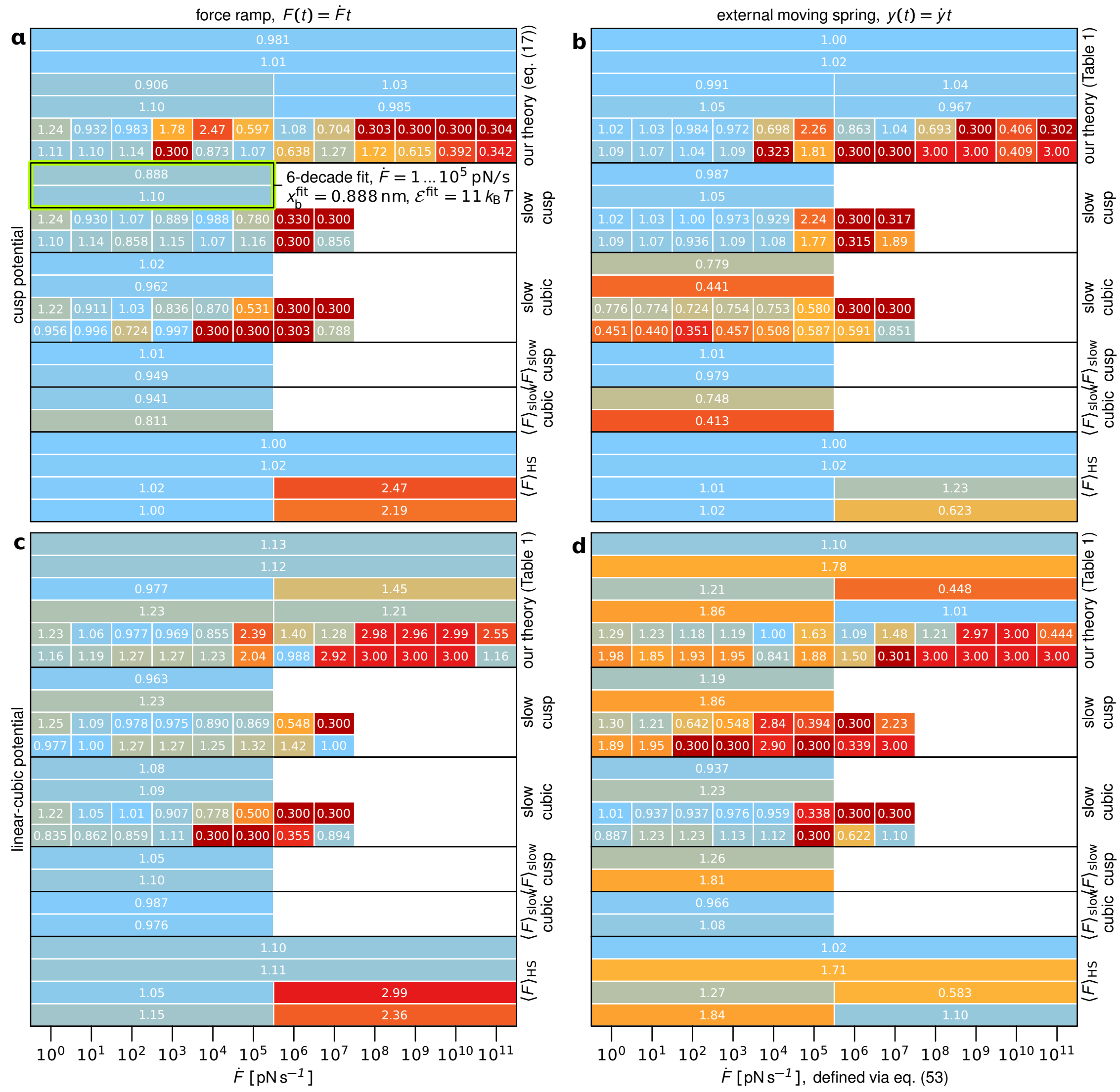

Supplementary Figure 1: Testing theories with Brownian dynamics simulations. To evaluate how well our theory fares in practice, we have performed Brownian Dynamics simulations of particles bound either via a cusp-shaped (**a**, **b**) or linear-cubic potential (**c**, **d**) and acted upon by an external field/soft spring (**a**, **c**) or by a stiff spring (**b**, **d**). The rupture force histograms thus obtained were analyzed "locally" (performing a different fit for each loading rate) and "globally" (performing a single fit for several decades in $\dot{F}$) using both our own theory and the best available steady-state ("slow") theories (specifically, "slow cusp/cubic" denotes the DHS [14] (**a**, **c**)/Maitra-Arya [17] model (**b**, **d**), evaluated either for a cusp-shaped or for a linear-cubic binding potential, see Supplementary Note 1 for further details). We have furthermore followed standard operating procedures by alternatively fitting the mean rupture force $\langle F \rangle$ as a function of loading rate, using the analytical results of the DHS [15] (**a**, **c**)/Maitra-Arya model (**b**, **d**), again specialized either to a cusp-shaped or linear-cubic binding potential, and using the numerical interpolation derived by Hummer & Szabo [19] ("HS") that extends to arbitrarily high loading rates. Each single fit is represented by a box split in half along its vertical axis, with the box width indicating the range of external loading rates taken into account and the two halves containing the best-fit value for the attraction range x_b^{fit}, measured in multiples of its true value $x_b = 1\,$nm and the best-fit value $\mathcal{E}^{fit}$ for the energy barrier, also measured in multiples of its true value $\mathcal{E} = 10\,k_B T$. As an example, we have highlighted in green in **a** the results of a 6-decade fit to simulations of a cusp-shaped binding potential under a linearly increasing external force field. The entry spans 6 decades, from 1 to $10^5\,$pN s^{-1} and belongs to the category "slow/cusp", indicating that a Maximum-Likelihood analysis of rupture force data obtained under 6 different loading rates has been performed, using the "cusp" ($\nu = 1/2$) DHS [14] model and yielding the following results: $x_b^{fit} = 0.888\,$nm, $\mathcal{E}^{fit} = 11\,k_B T$.

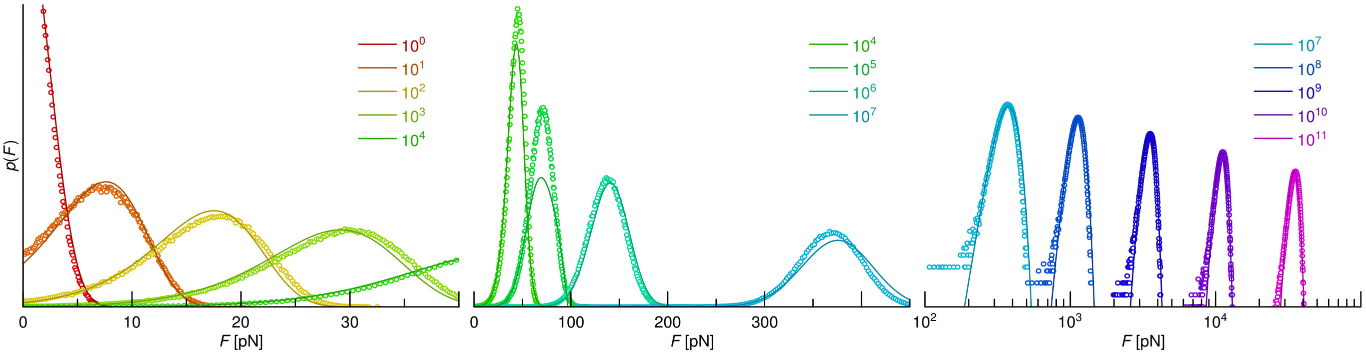

Supplementary Figure 2: Rupture force histograms for a linear-cubic binding potential. Using a linear-cubic binding potential $U(x) = \mathscr{E}(x/x_\mathrm{b})^2 \times (3-2x/x_\mathrm{b})$, we have generated rupture force histograms analogous to those shown in fig. 3 in the main text. Solid lines show a global fit of our theory with μ as a free parameter (see Table 1). The best-fit parameters thus obtained are $(\mathscr{E} = 11.2\,k_\mathrm{B}T, x_\mathrm{b} = 1.13\,\mathrm{nm}, D = 774\,\mathrm{nm}^2\,\mathrm{s}^{-1}, \mu = 5.75)$

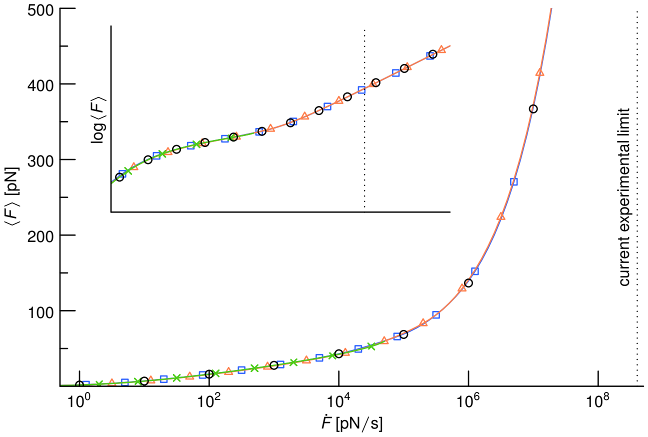

Supplementary Figure 3: Mean rupture forces for a linear-cubic binding potential. Circles: mean rupture force $\langle F \rangle$ as determined from our numerical simulations, using a linear-cubic binding potential $U(x) = \mathscr{E}(x/x_\mathrm{b})^2 \times (3-2x/x_\mathrm{b})$ (see Supplementary Fig. 2). Crosses: DHS [14] fit, with fit parameters ($\mathscr{E} = 9.76\,k_\mathrm{B}T, x_\mathrm{b} = 0.99\,\mathrm{nm}, D = 744\,\mathrm{nm}^2\,\mathrm{s}^{-1}$). Triangles: Hummer-Szabo [19] fit, with fit parameters ($\mathscr{E} = 11.1\,k_\mathrm{B}T, x_\mathrm{b} = 1.10\,\mathrm{nm}, D = 772\,\mathrm{nm}^2\,\mathrm{s}^{-1}$). Squares: mean rupture force $\langle F \rangle$ determined from $p(F)$ (see Methods section), using the fit parameters obtained in Supplementary Fig. 2. Inset shows the same data in double-logarithmic coordinates.

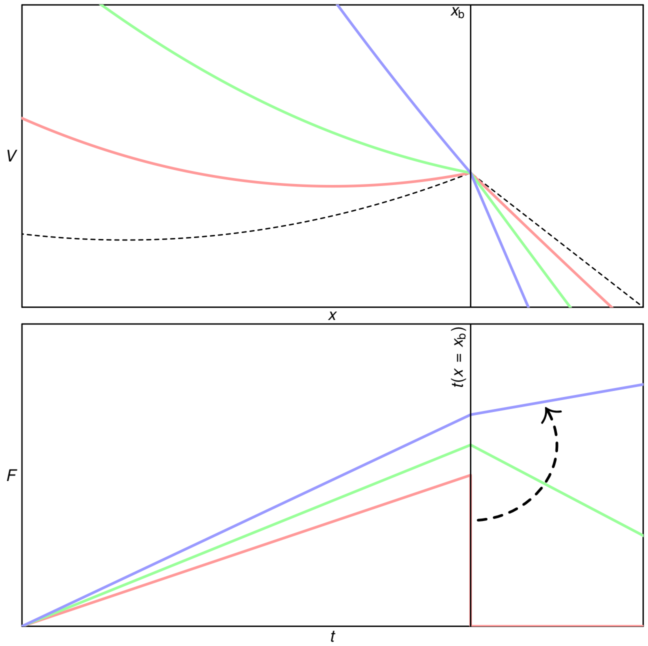

Supplementary Figure 4: Generalized working definition of bond rupture. At low pulling speeds (red curve), the bound state remains stable up to the time of bond rupture; on an experimental timescale, the subsequent decrease in pulling force appears instantaneous. Within the ballistic regime, the bound state is no longer stabilized by a free energy barrier; still, a pronounced change in intermolecular binding forces $-U'(x \approx x_b)$ manifests itself in a force maximum at x_b for intermediate pulling speeds ($\gamma v - \kappa[y(t_{\text{rupture}}) - x_b] - F_R < 0$, green curve), whereas at even larger pulling speeds the force continues to rise towards its ultimate limit $F(t \to \infty) = v\gamma - F_R$, but exhibits a kink at $x = x_b$ (blue curve). Hence, the characteristic signature of bond rupture is found to vanish gradually beyond the critical pulling force, disappearing completely only in the ultimate limit $v = \infty$.

Supplementary Note 1: Benchmarking. To assess the practical performance of our theory in comparison to established models of dynamic force spectroscopy, we generate synthetic rupture force histograms over a wide range of pulling speeds ranging from 1 to $10^{11}\mathrm{pN\,s^{-1}}$ (using a direct integration of the underlying Langevin equation, as explained in the Methods section of the main text). In addition to the cusp-shaped binding potential described in the main text, we also consider a linear-cubic binding potential $U(x) = \mathscr{E}(x/x_\mathrm{b})^2 \times (3 - 2x/x_\mathrm{b})$. In contrast to the cusp potential, the linear-cubic potential lacks an absorbing boundary, such that rebinding must be prevented by strong repulsive forces within the unbound state. We thus only consider the bond broken once x has passed $1.5x_\mathrm{b}$. For each of the four different experimental setups (cusp/field, linear-cubic/field, cusp/spring, linear-cubic/spring), we obtain a set of 12 different rupture force histograms, each of which we first analyze separately. Apart from our own theory, we also apply the corresponding results by DHS [14] and Maitra & Arya [17] (and eq. (4-S)), depending on which best applies to the experimental setup at hand.

Furthermore, we generate "global" fits by subsuming histograms obtained under a range of different pulling speeds and analyzing them all at once using the maximum-likelihood method proposed in [25]. Since we do not want to bias the results by our choice of starting value, we use a global optimization method (the `NMinimize` optimizer integrated into *Mathematica*), only restricting the range of allowed parameters to a rather large region ($3k_\mathrm{B}T < \mathscr{E} < 30k_\mathrm{B}T$, $0.3\,\mathrm{nm} < x_\mathrm{b} < 3\,\mathrm{nm}$, $300\,\mathrm{nm^2\,s^{-1}} < D < 3000\,\mathrm{nm^2\,s^{-1}}$, $0.3 < \mu < 9$). For each fit, "global" and "local", 1600 measured rupture forces per loading rate were used for the analysis.

Conventionally, global fits are often obtained not by fitting the rupture force distributions themselves, but by analyzing the mean rupture force $\langle F \rangle$ [39] (or the most probable rupture force [13]) as a function of the external loading rate. We have thus included this method as well, but only using the conventional (quasistatic) expressions for $\langle F \rangle$ [15, 17] and the extrapolation by Hummer & Szabo [19], though one may derive analogous results from our theory, if so desired (see the Methods section of the article).

Supplementary Fig. 1 provides an overview of the fit parameters thus obtained and their relative deviations from the true model parameters.

Supplementary Note 2: Microscopic Kramers rate for a cusp potential in stiff spring limit. In [17], the work of DHS [14] and Friddle [15] is extended to explicitly account for a harmonic force transducer, thus relaxing the commonly made assumption that the pulling device is much softer than the intramolecular bond. Only a linear-cubic binding potential (*i.e.* the $\nu = 2/3$ case in [14]) is considered in [17], but not the $\nu = 1/2$ "cusp" scenario. To compare our results to the optimized version of the conventional steady-state approximation, we provide here a short derivation of a "cusp-optimized" counterpart to the results given in [17]. The thermal escape of a particle, moving in an energy landscape

$$V_\mathrm{total}(x,t) = U(x) + V(x,t) \equiv U(x) + \frac{1}{2}\kappa[x - y(t)]^2\,, \tag{1-S}$$

$$U(x) = \begin{cases} \mathscr{E}(x/x_\mathrm{b})^2\,, & x < x_\mathrm{b} \\ -\infty, & \text{otherwise} \end{cases}\,, \tag{2-S}$$

over an effective energy barrier of height $\chi\beta\mathscr{E} \gg 1$, $\chi = 1 + \kappa x_\mathrm{b}^2/2\mathscr{E}$, can be described using Kramers theory [14, 26], where the rate of escape is given by

$$k(F) = D\left(\int_\mathrm{well} \mathrm{e}^{-\beta V_\mathrm{total}(y,t)}\,\mathrm{d}y \int_\mathrm{barrier} \mathrm{e}^{\beta V_\mathrm{total}(z,t)}\,\mathrm{d}z\right)^{-1} \approx k_0\chi^{3/2}\left[1 - \frac{F}{F_\mathrm{c}}\right]\mathrm{e}^{\beta\mathscr{E}[1-\chi(1-F/F_\mathrm{c})^2]}\,, \tag{3-S}$$

$F(t) = \kappa y(t)/\chi$, $F_\mathrm{c} = 2\mathscr{E}/x_\mathrm{b}$ and k_0 is the associated Kramers rate in absence of the force transducer. The rate expression above can be used to compute the accompanying RFD via eq. (1). For a force ramp, *i.e.* $y(t) = \dot{y}t$ and thus $F(t) = \dot{F}t$ with $\dot{F} = \mathrm{const}$, the resulting RFD reads

$$p(F) = \frac{k(F)}{\dot{F}}\exp\left(-\frac{1}{\chi\beta\dot{F}x_\mathrm{b}}\left[\frac{k(F)}{(1 - F/F_\mathrm{c})} - k(0)\right]\right)\,. \tag{4-S}$$

Finally, the mean and the variance of the distribution can be deduced along the lines of [15],

$$\langle F\rangle \approx F_{\mathrm{c}}\left(1-\left[1-\frac{\mathrm{e}^{qX}\,\mathrm{E}_1(qX)}{\chi\beta\mathscr{E}}\right]^{1/2}\right), \tag{7a-S}$$

$$\sigma_F^2 \approx \frac{\pi^2}{6}\left(\frac{1}{\chi\beta x_{\mathrm{b}}(1+qX)}\right)^2\left(1-\frac{\mathrm{e}^{qX}\,\mathrm{E}_1(qX)}{\chi\beta\mathscr{E}}\right)^{-1}, \tag{7b-S}$$

where q, X and $\mathrm{E}_1(z)$ are defined as in the Methods section of the main text.

Supplementary Note 3: Experimental detection of "ballistic rupture events". During the peer-review process the question was raised as to how one might measure, or even define, a "rupture" event when there is no longer an effective free energy barrier to stabilize the bound state. A step-like transition between two discrete, stable equilibrium positions (*i.e.*, the time-dependent energy minimum $\langle x\rangle$ within the bound state and the location $y(t)$ of the external force actuator) can arise only from the metastable free energy landscape pertaining to subcritical pulling forces $F < F_{\mathrm{c}}$. Yet, even as the effective free energy barrier vanishes, the intramolecular binding potential U still underlies the combined effective potential $U+V$, and with it the strong variation in intramolecular forces $F(x) = -U'(x)$ characteristic of a well-defined transition region around x_{b}. The question of how to best relate experimentally measured force traces to the first-passage time distribution at x_{b} is an (albeit important) implementation detail to be decided by the practitioner. Still, to illustrate the matter and to provide at least one possible working definition of "ballistic bond rupture", we consider a non-singular, but sharp transition region characterized by finite slopes $-U'(x\uparrow x_{\mathrm{b}}) = F_{\mathrm{L}}$, $-U'(x\downarrow x_{\mathrm{b}}) = F_{\mathrm{R}}$. Evaluating the athermal dynamics of $x(t)$,

$$\gamma\dot{x}(t) = \kappa[y(t)-x(t)] + F_{\mathrm{R}} \qquad \text{for } x > x_{\mathrm{b}}\,, \tag{8a-S}$$

$$\begin{aligned} -\gamma\delta\dot{x}(t) &\equiv -\gamma[\dot{y}(t)-\dot{x}(t)] \\ &= \kappa[y(t)-x(t)] + F_{\mathrm{R}} - \gamma\dot{y}(t) \\ &\equiv -\gamma v(t) + F_{\mathrm{R}} + \kappa\delta x(t)\,, \end{aligned} \tag{8b-S}$$

we find that, as we enter the ballistic regime (*i.e.*, the pulling force at the time of rupture exceeds $F_{\mathrm{c}} = F_{\mathrm{L}}$), a friction-limited dynamic equilibrium ensues, with the steady-state extension of the external force actuator saturating at $F = \gamma v - F_{\mathrm{R}}$. Depending on F_{R}, this implies either a force maximum at $x = x_{\mathrm{b}}$ or an ongoing increase in the pulling force that is, however, preceded by a detectable kink as x crosses the barrier position.

Hence, the characteristic "rupture signature" seen in the time-resolved pulling force $F(t)$ does not disappear immediately as loading rates increase beyond the critical loading rate, but instead vanishes gradually (as sketched in Supplementary Fig. 4), thus in principle allowing for experimental detection for all but infinite loading rates $v = \infty$.
