# Supplementary material for: Theory of rapid force spectroscopy
Source: Nat Commun. 2014 Jul 31;5:4463. doi: 10.1038/ncomms5463 (PMC4124868; doi:10.1038/ncomms5463)
Supplement: Supplementary Data 1 — Mathematica Notebook for data analysis and User's Guide accompanying Mathematica Notebook [file ncomms5463-s2.zip › supplementary_data_1/User's Guide.pdf]

User's guide for

`Maximum Likelihood Analysis.nb`

The supplied *Mathematica* notebook `Maximum Likelihood Analysis.nb` provides a convenient graphical user interface to the Maximum Likelihood analysis technique described in [25] and in the Methods of the main text. It consists of a single cell that can be evaluated by clicking on it and pressing "shift" + "enter", or by selecting "Evaluation" > "Evaluate Notebook" from the menu bar. This will produce a graphical interface at the bottom of the notebook, see Fig. 1. Experimental data can be imported using the "+" button and should either come in the form of a rupture force histogram, *i.e.* a whitespace-delimited table of forces F and corresponding rupture force probabilities $p(F)$, as in the following example:

```
0       0
0.5     0.2
1       0.6
1.5     0.1
2       0.05
2.5     0.05
...
```

or in the form of a list of experimentally observed rupture forces, *i.e.* something along the lines of

```
0.9
1.8
1.1
0.95
0.6
1.3
...
```

Note that scientific notation can be used as long as it adheres to the C/Fortran convention (*e.g.* 4×10^5 = `4e5` or `4E5`). To check whether the data has been interpreted

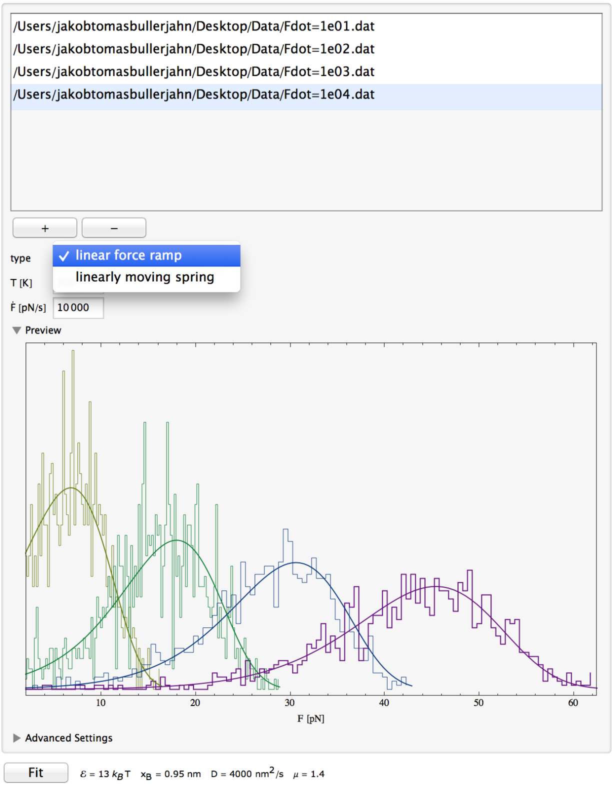

Figure 1: Preview window. For every data set, the experimental setup must be selected from a drop down menu and the associated protocol parameters must be entered in the appropriate input fields. The preview option allows the user to compare her data to the theoretical model.

correctly, the "Preview" panel can be used to quickly visualize the given data (if supplied as a list of rupture forces, a 100-bin rupture force histogram will be shown). After importing your data, select each data set in turn and specify a loading protocol (either a linearly moving spring or a linearly increasing external force) by selecting the appropriate entry from the drop-down menu and entering the relevant parameters (loading rate and temperature for linear force ramps, velocity, stiffness and temperature for linearly moving springs). Per default, the rupture force is determined at the very instant of bond breaking, corresponding for the spring protocol to the Hummer-Szabo [19] convention, *i.e.*, $F^* = F$ in Table 1. Alternatively, the pulling force may be averaged over quasistatically as proposed by Maitra and Arya [17] by selecting "moving average" from the corresponding drop down menu (see Fig. 2). A fit can then be obtained by pressing the "Fit" button; ordinarily, a solution is sought within the following parameter range:

$$
\begin{aligned}
1 &< \mathscr{E}\,[k_\mathrm{B}T] < 30 \\
0.2 &< x_\mathrm{b}\,[\mathrm{nm}] < 5 \\
100 &< D\,[\mathrm{nm}^2/\mathrm{s}] < 10000, \\
0.3 &< \mu < 9.
\end{aligned}
$$

Different parameter bounds as well as the choice of an optimization algorithm can be specified in the "Advanced Settings" panel, see Fig. 3. Note that the fit might take several minutes, during which *Mathematica* may become unresponsive. If the allowed parameter range is very large, it may even be necessary to increase the number of initial search points or the maximum number of iterations to achieve convergence. Once a fit is obtained, the quality of the fit can be inspected visually in the preview panel (also shown in Fig. 1).

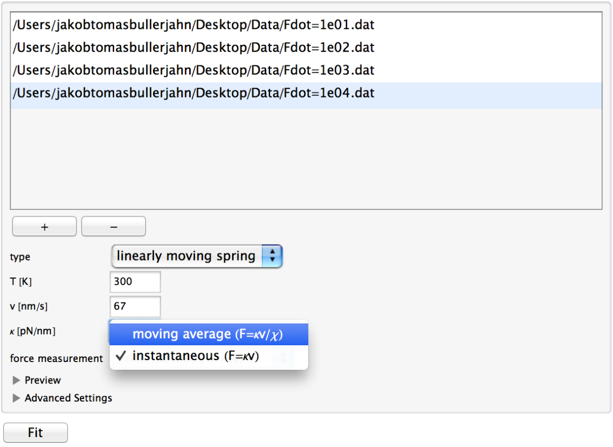

Figure 2: Choosing the correct force definition. For a moving external spring, rupture forces can be evaluated either at the exact moment of rupture or as a time-moving average (cf. refs. [17, 19] and eq. (53)).

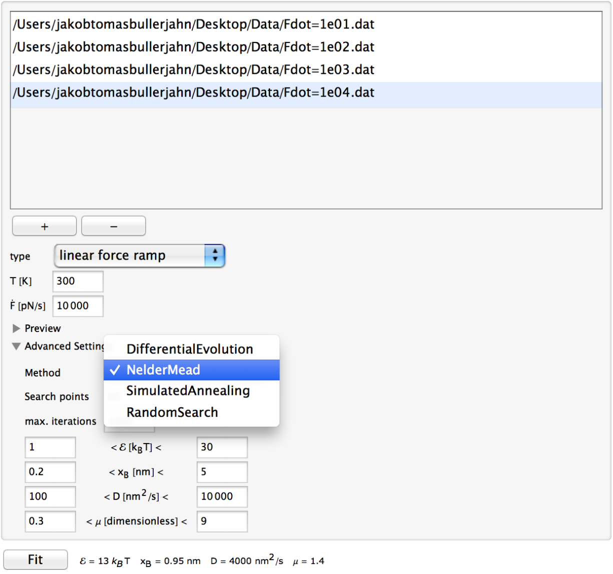

Figure 3: Customizing the fit procedure. Using the "Advanced Settings", the user can choose between four different optimization methods and can alter various parameters concerning the fit.
